# Exploring the sequence variability of polymerization-involved residues in the production of levan- and inulin-type fructooligosaccharides with a levansucrase

**DOI:** 10.1038/s41598-019-44211-5

**Published:** 2019-05-22

**Authors:** Christian Possiel, Maria Elena Ortiz-Soto, Julia Ertl, Angela Münch, Andreas Vogel, Ramona Schmiedel, Jürgen Seibel

**Affiliations:** 1 Institute of Organic Chemistry, University of Würzburg, Am Hubland, 97074 Würzburg, Germany; 2 C-LEcta GmbH, Perlickstr. 5, 04103 Leipzig, Germany

**Keywords:** Molecular engineering, Biocatalysis

## Abstract

The connection between the gut microbiome composition and human health has long been recognized, such that the host-microbiome interplay is at present the subject of the so-called “precision medicine”. Non-digestible fructooligosaccharides (FOS) can modulate the microbial composition and therefore their consumption occupies a central place in a strategy seeking to reverse microbiome-linked diseases. We created a small library of *Bacillus megaterium* levansucrase variants with focus on the synthesis of levan- and inulin-type FOS. Modifications were introduced at positions R370, K373 and F419, which are either part of the oligosaccharide elongation pathway or are located in the vicinity of residues that modulate polymerization. These amino acids were exchanged by residues of different characteristics, some of them being extremely low- or non-represented in enzymes of the levansucrase family (Glycoside Hydrolase 68, GH68). F419 seemed to play a minor role in FOS binding. However, changes at R370 abated the levansucrase capacity to synthesize levan-type oligosaccharides, with some mutations turning the product specificity towards neo-FOS and the inulin-like sugar 1-kestose. Although variants retaining the native R370 produced efficiently levan-type tri-, tetra- and pentasaccharides, their capacity to elongate these FOS was hampered by including the mutation K373H or K373L. Mutant K373H, for instance, generated 37- and 5.6-fold higher yields of 6-kestose and 6-nystose, respectively, than the wild-type enzyme, while maintaining a similar catalytic activity. The effect of mutations on the levansucrase product specificity is discussed.

## Introduction

The French gastronome Jean Anthelme Brillat-Savarin wrote in his Physiologie du Gout: “Dis-moi ce que tu manges, je te dirai ce que tu es” (Tell me what you eat and I will tell you what you are). Almost two centuries later the connection between the intestinal microbiome composition and human digestive and mental health continues to amaze researchers^1^. The microbiome makes up to 100 trillion cells, ten-fold more than cells in an higher eukaryote host^2^. The proliferation of beneficial bacteria known as probiotics (i.e. bifidobacteria and lactobacilli) can be modulated by the dietary consumption of prebiotics, which is often accompanied by a decline in the number of pathogenic species. Anxiolytic and antidepressant effects are among the major benefits for the host well-being that have been associated with the metabolism of probiotics^1,3^. With the advent of the ‘omics era, the treatment for microbiome-linked diseases pursues a personalized care, increasing the demand for pre- and probiotics. In consequence, the market for prebiotics and other functional food ingredients has kept a brisk pace over the last three decades, and is poised to exceed USD 750 million by 2024, stimulated by concerns towards diabetes, obesity and digestive health (https://www.gminsights.com/). Inulin (^1^F), a well-known functional ingredient is predominantly isolated from chicory and can be hydrolyzed to obtain mixtures of products of lower degree of polymerization (DP)^4^. Short chain ^1^F-type FOS have half the calories per gram than traditional sweeteners, being thus attractive as food additives in low-caloric products^5^. Trisaccharides 1- and 6-kestose as well as the tetrasaccharide nystose and oligosaccharides of the neo-series possess bifidogenic activity^6,7^.

Inulin and levan (^6^F) contain β2-1 and β2-6 linkages, respectively, and are synthesized by enzymes from families GH32 and GH68 using sucrose as starting material. While ^1^F-type FOS are well studied and are commercialized by different companies, ^6^F-type products such as 6-kestose and 6-nystose are less common. Some enzymes from family GH32, i.e. fructofuranosidases from yeast *Schwanniomyces occidentalis* or an invertase from *Saccharomyces cerevisiae* have been successfully used in the synthesis of ^6^F-type oligosaccharides^7–9^. However, most levansucrases from family GH68 synthesize polymers larger than 2 × 10^6^ Da, and some of them produce a mixture of polymer and FOS^10–12^. 6-kestose is the first product of levansucrase-catalyzed transfructosylation reactions and is as well the best acceptor of the fructosyl-moiety during polymerization. Accordingly, it does not accumulate in substantial amounts during the reaction course, this also being the case for other short FOS with β2-6 linkages.

An alternative strategy to obtain FOS from levan involves the application of endo-levanases. High- and low molecular weight levans of bacterial and plant origin can be hydrolyzed at different rates to produce FOS of DP ≤ 13 employing the endo-levanase BT1760 of the gut resident *Bacteroides thetaiotaomicron*^13^. In a similar approach, a protein fusion of *Bacillus subtilis* levansucrase and an endo-levanase from *Bacillus licheniformis* can be applied for the simultaneous synthesis and hydrolysis of levan to generate FOS of DP 2 to 10^14^.

A number of *in vitro* studies showed that many species of *Bifidobacteria*, one of the major bacterial genera in mammalian microbiomes, utilize FOS of DP 2–8 rather than larger oligosaccharides^15^. Therefore, the homogeneity of FOS preparations regarding their DP is of considerable interest. Here we report the creation of a library of *B*. *megaterium* levansucrase variants with the capacity to synthesize predominately FOS of DP 2–8, in contrast to the wild-type enzyme that produces low amounts of FOS with DP 2–20^16,17^. The levansucrase library explores the modification of residues R370, K373 and F419, which are situated in the central sucrose/oligosaccharide binding pocket. Analysis of the crystal structures of *B*. *subtilis* (PDB 1pt2) and *B*. *megaterium* (PDB 3om2) levansucrases shows that the side chain of residue F419 (F409 in *B*. *subtilis* levansucrase) is partially buried and therefore not expected to engage with elongating FOS via CH–π interactions. Exchange of F419 by other amino acids might, however, have an impact in the orientation of the side chains of adjacent residues R370 and Y421, both known for their participation in product binding and control of the hydrolysis/transfer partition^16,18^. Substitution of residue F419 could thus indirectly provoke changes in the +1 and +2 subsites.

The role of residues R370 and K373 in the modulation of the polymerization process is well established^18–20^. These positions were revisited in this work to allow substitutions previously unexplored, and to expand the range of possible amino acid combinations. By diminishing the levansucrase ability to produce larger products, we anticipated the accumulation of short-length chain oligosaccharides, in particular 6-kestose and 6-nystose, whose biological activity requires a more in-depth study.

Single variant K373L was employed for the synthesis of 1-kestose, 6-kestose and 6-nystose, which were received with a purity higher than 95%.

## Results and Discussion

### Creation of a library of *B*. *megaterium* levansucrase variants

**Figure 1:**
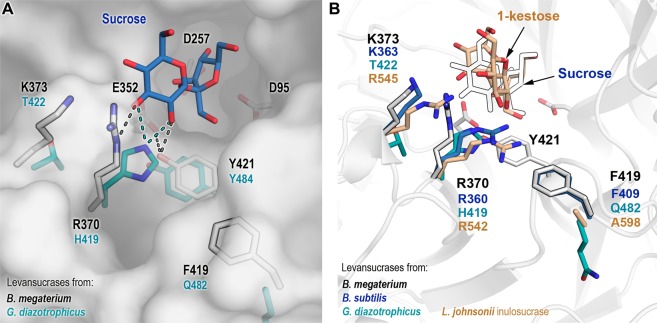
Modified positions in *B*. *megaterium* levansucrase library. (**A**) Selected positions for mutagenesis in *B*. *megaterium* levansucrase are displayed as outlined white sticks. Catalytic amino acids and residue Y421, which has a pivotal role in hydrolysis and transfer, are shown as white sticks. Corresponding residues in the levansucrase from Gram-negative *Gluconacetobacter diazotrophicus* are depicted in cyan. Possible hydrogen bond interactions with other residues and with sucrose are shown as dotted lines. Sucrose (blue) was aligned from the crystal structure of *B*. *subtilis* levansucrase (PDB 1pt2). (**B**) Residues found at analogous positions in the inulosucrase from *L*. *johnsonii* (PDB 2yft, orange) and in the levansucrases from *B*. *megaterium* (PDB 3om2, white sticks), *B*. *subtilis* (PDB 1pt2, blue) and *G*. *diazotrophicus* (PDB 1w18, cyan). Equivalent positions to Y421 are not shown in the right panel for the sake of clarity. Numbering corresponding to *B*. *megaterium* levansucrase is displayed in black, and for the other enzymes according to their color code.

A library of *B*. *megaterium* levansucrase variants was designed to introduce changes in positions R370, K373 and F419 (Table 1). These residues are located in two of the eight loops that encompass the catalytic pocket (Fig. 1) of *B*. *megaterium* levansucrase. Residues R370 and K373 constitute some of the earlier binding subsites in the path of polymer elongation and their role in defining the oligosaccharide product size has been demonstrated with the levansucrase from *B*. *megaterium* and other enzymes from family GH68^10,18,19,21^ (Supplementary Information, Fig. 1). R370 is a semi-conserved residue that is substituted by histidine in GH68 enzymes from Gram-negative bacteria. It is situated in the +1 subsite and forms hydrogen bonds with the 2- and 3-OH groups of the glucosyl-moiety of the donor sucrose (Fig. 1). Consequently, changes at this position reduce the rate of formation of the covalent intermediate fructosyl-enzyme, while leaving the hydrolyzing activity unaffected and favoring the synthesis of di- and trisaccharides^21^.

**Table 1:** Library of *B*. *megaterium* levansucrase variants.

Position	Mutation	Theoretical number of clones (Library size)	Number of screened clones
R370	NDEHQRV	280	768
K373	HKRLS		
F419	FQASWEDY

K373 and F419 are found in hypervariable regions that tolerate amino acids of diverse biochemical properties (Supplementary Information, Table S1). Threonine and lysine are the most represented residues at position 373, with arginine, methionine and other amino acids also being observed in the Multiple Sequence Alignment (MSA) of 372 non-redundant sequences of GH68 enzymes. Mutation of residue K373 by alanine and arginine in *B*. *megaterium* levansucrase restricts the formation of FOS of DP > 6 and DP > 8, respectively^19^.

The MSA shows that residue F419 is replaced by glutamine or glutamic acid in GH68 members from Gram-negative bacteria, while alanine, phenylalanine, tryptophan or serine are permitted in levansucrases and inulosucrases from Gram-positive organisms. Residues with aromatic side chains can interact to different extents with carbohydrates in stacking geometry via CH–π interactions^22^. As the side chain of F419 is partially buried, such contacts are possibly not established. However, modification of F419 could alter the fine-tuned protein-ligand contacts in the +1 and +2 subsites. F419 is found in the vicinity of Y421, the latter being able to form hydrogen bonds with general acid/base E352 and the 2-OH group of the glucosyl-moiety in the +1 subsite^10,16^. F419 is also close to residue R370, which is involved in sucrose/FOS binding. Hence, modifications in this region may result in changes in the product pattern of *B*. *megaterium* levansucrase. A structural alignment of the inulosucrase from *Lactobacillus johnsonii*, the fructosyltransferase 6-SST/6-SFT from *Pachysandra terminalis* and the levansucrases from *B*. *subtilis* and *B*. *megaterium* reveals important differences in the enzyme-acceptor binding mode (Supplementary Information, Fig. 1). Due to structural differences between inulin and levan (the former containing fructose residues bound through β2-1 linkages and the latter via β2-6 connections), residue R370 seems to interact with ^6^F-type FOS only at the +1 subsite^23,24^. Equivalent residue R542 in the inulosucrase from *L*. *johnsonii* is involved in hydrogen bonds with the glucosyl-moiety (+1 subsite) of sucrose when it acts as donor (Supplementary Information, Fig. 1A), and with the glucosyl-unit found at the +2 subsite in the ^1^F-type acceptor molecule 1-kestose^25^ (Supplementary Information, Fig. 1B). Residues 370, 373 and 419 were therefore selected with the aim of exploring the functional combination of amino acids with diverse side chains (Table 1) at these positions and the effect of mutations in both, product size and enzyme’s regioselectivity.

### Selection and analysis of variants

*Escherichia coli* BL21 cells were transformed with a plasmid derivative from pRSF (Novagen) containing *B*. *megaterium* levansucrase library. Cleared-cell lysates of 768 clones were screened for global activity (hydrolysis and transfer) on 0.5 m sucrose through the detection of reducing sugars after 10 min incubation at 37 °C. The product profile of active variants was analyzed on TLC after incubation of 1 µL cleared-cell lysates with 0.5 m sucrose for 20 h (Supplementary Information, Fig. S2). Variants synthesizing or accumulating smaller products than the parental levansucrase were selected. Levan and large FOS were detected on TLC as products with low mobility that did not migrate from the sample application spot. Three variants producing similar amounts of large FOS/levan than the wild-type enzyme were also chosen with the aim of exploring whether a different combination of amino acids would result in the wild-type behavior (Supplementary Information, Fig. S2). Thirty clones were selected based on these criteria and sequenced. Sixteen unique single, double and triple variants were identified and further characterized (Table 2). Besides the expressed levansucrase, *E*. *coli* BL21 does not produce other enzymatic activities able to transform sucrose or FOS. Hence, reactions for product analysis by HPAEC-PAC were carried out using cleared extracts, while purified proteins were applied for determination of kinetic parameters. For short, variants were labeled according to their position in 96 well plates during screening. As expected, all variants that included a mutation at residue R370 or K373 synthesized FOS with DP < 8, in contrast to the wild-type levansucrase that was able to produce FOS of DP 2-20 (Table 2). Single variant F419Y showed a similar behavior to the wild-type enzyme (Supplementary Fig. S3), which may indicate that the tyrosine OH-group did not create new interactions with ^1^F-type products or affected the orientation of R370 side-chain regarding to donor/acceptor molecules.

**Table 2:** Characterization of *B*. *megaterium* levansucrase variants.

	Products size	Variant	Position			K_M_ (mm)	k_cat_ (s−1)	k_cat_/K_M_ (s^−1^ m**m**^−1^)
			370	373	419			
Group 1	DP 2–20	WT^a^	R	K	F	10.8 ± 1.5	197.9 ± 4.0	18.32
		3A6	R	K	**Y**	—	—	—
Group 2	DP 4	1H11	**H**	K	F	—	—	—
		2B12	**H**	**S**	F	—	—	—
		7D12^b^	**H**	K	**W**	—	—	—
Group 3	DP 8	1G2	R	**R**	F	—	—	—
		2H11	R	**R**	**Y**	—	—	—
		3H5	R	**R**	**W**	—	—	—
Group 4	DP 4	2A5	**Q**	K	F	199.4 ± 15.2	42.7 ± 2.4	0.21
		5F11	**Q**	K	**W**	—	—	—
		3G5	**Q**	K	**Y**	—	—	—
		3E7	**Q**	**R**	**W**	114.8 ± 9.1	24.0 ± 0.9	0.21
Group 5	DP 6–7	1B1	R	**H**	F	41.8 ± 6.6	197.5 ± 8.4	4.72
		2E11	R	**H**	**Y**	—	—	—
		3B9^b^	R	**H**	**E**	—	—	—
		3C5	R	**L**	F	12.5 ± 1.3	122.5 ± 2.3	9.80
		4E1	R	**L**	**Y**	—	—	—

Mutated residues are shown as underlined bold letters. Catalytic parameters were only calculated for representative variants of groups 4–5. Reactions were performed in 50 mm Sörensen buffer pH 6.5, containing varying concentrations of sucrose and purified levansucrase variants. Variants not kinetically characterized are indicated by a horizontal dash (−).

^a^From^16^.

^b^These variants display very low activity and poor sucrose conversion, producing mainly di- and trisaccharides.

Based on the mutated position and their product pattern, variants were categorized in 5 groups. Group 1 contains variant F419Y, which reproduces the wild-type performance. Variants from group 2 carry the mutation R370H and may include substitutions at residues K373 and F419. Variants in group 3 maintain the mutation K373R with or without exchange of F419. All variants in group 4 possess the mutation R370Q and may contain mutations at amino acids K373 and F419; and finally, group 5 includes variants carrying the modification K373H or K373L, with or without substitution of F419. Enzymes producing high yields of short chain oligosaccharides were kinetically characterized.

Tyrosine and tryptophan were frequently found in active variants carrying a mutation at residue F419, and both substitutions had a rather small influence on the product distribution. It is plausible that the substitution of phenylalanine by the bulkier tryptophan could introduce steric hindrance between residues R370H and F419W, thus decreasing drastically the activity of variant 7D12. Glutamic acid was also found at position 419 (variant 3B9); however, this exchange resulted in a nearly inactive variant. Alanine is found at the equivalent position in the inulosucrase from *L*. *johnsonii* (PDB 2yft), while glutamine is found at this position in the levansucrases from *Erwinia amylovora* (PDB 4d47) and *G*. *diazotrophicus* (PDB 1w18) (Fig. 1). The side chain of equivalent amino acids in these two enzymes is oriented towards the protein’s core. Conversely, in *B*. *subtilis* and *B*. *megaterium* levansucrases the side chain of phenylalanine (F409 in the former) is directed toward the oligosaccharide binding surface and is partially exposed. Although some orientations of the glutamic acid side chain (rotamers) could theoretically be tolerated at position 419, some other may induce a major disturbance of adjacent loops (Supplementary Information, Fig. S4).

**Figure 2:**
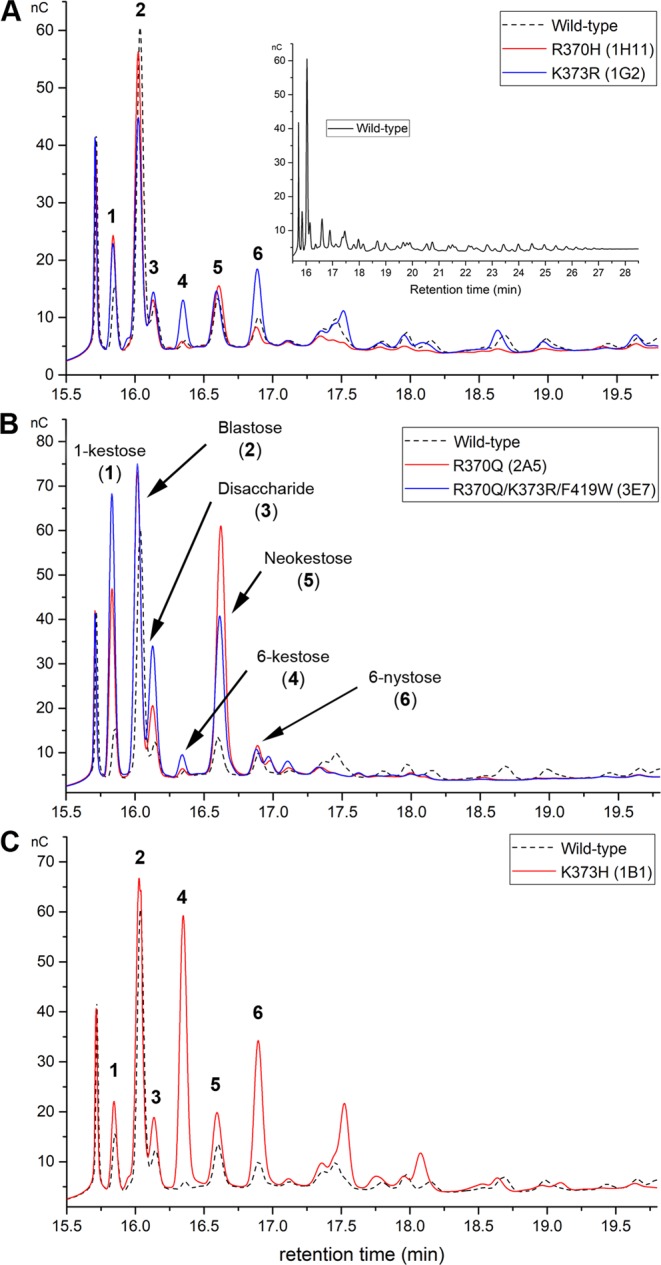
HPAEC-PAC analysis of the product specificity of *B*. *megaterium* levansucrase variants. (**A**) FOS profile of variants R370H and K373R (from groups 2 and 3); (**B**) FOS produced by variants R370Q and R370Q/K373R/F419W (from group 4); and (**C**) FOS synthesized by variant K373H (from group 5). Products of the wild-type levansucrase (group 1) are shown as black dotted lines. Reactions were performed in Sörensen buffer using 2 U mL^−1^ of each enzyme and 0.5 m sucrose. The products were analyzed after 24 h reaction, at comparable sucrose conversions for all variants (around 90–95%). This figure is composed of cropped chromatograms focused on the oligosaccharide products of variants; full-length chromatograms are included as Supplementary Information, Figs S5 and S6.

The size of products synthesized by variants from groups 2 and 3 was reduced to DP 4 and DP 8, respectively. These results are in agreement with data reported for *B. subtilis* levansucrase single variant R360H^21^ (R370H in this publication) and *B. megaterium* levansucrase K373R^19^. Variants from these two groups synthesized short FOS inefficiently, with mutant K373R (1G2) producing FOS of DP 4-8 in similar yields to those of the wild-type enzyme (Fig. 2A). Variants R370H and K373R synthesized some products in a similar fashion. Mutant R370H (1H11) produced preferentially 1-kestose, blastose, a disaccharide of unknown structure, neokestose (containing a fructosyl-moiety with β2–6 linkage to the glucose residue of sucrose) and 6-nystose, while variant K373R synthesized in addition 6-kestose and small amounts of larger FOS up to DP8. Because the effect of mutations R370H and K373R were previously reported in various levansucrases, analysis of these variants was not pursued.

Variants from groups 4 and 5 produced di-, tri- and tetrasaccharides more efficiently than other variants or the parental levansucrase (Fig. 2B,C). These products were accumulated over the reaction course along with blastose, which was the primary product of all variants and the wild-type enzyme (Supplementary Information, Figs S7 and S8).

Group 4 variants, which contain a glutamine at position 370 synthesized preferentially 1-kestose, neokestose and blastose (Fig. 2B). Changes at this position have been extensively explored in various enzymes from family GH68, in particular the exchange by histidine (or the corresponding substitution of histidine by arginine in GH68 enzymes from Gram-negative bacteria), leucine, alanine, and serine. These mutations resulted in a prominent increase in hydrolysis, low yields of transfructosylation products and inability to synthesize FOS larger than DP 4–5^10,18,21,26^. The equivalent mutation R296Q in *Zymomonas mobilis* levansucrase increased the amount of tri-, tetra- and pentasaccharides^27^.

Being good acceptors to produce larger FOS, 6-kestose and 6-nystose are usually not observed over the reaction course of *B*. *megaterium* wild-type levansucrase. Albeit the amounts of 6-kestose and 6-nystose synthesized by mutant R370Q were similar to those of the wild-type enzyme, this variant did not produce oligosaccharides beyond DP 4 (Supplementary Information, Fig. S8), suggesting that substitution of arginine 370 by glutamine abated the variant’s capacity to synthesize and elongate efficiently ^6^F-type sugars (Variant 2A5, Fig. 2B). Mutation R370Q is inferred to modify the size and charge of the +1/+2 binding subsites. Asparagine at this position can probably stabilize sucrose conformations (when acting as acceptor of the fructosyl-residue) leading to the synthesis of 1-kestose and neokestose in a more efficient fashion than arginine. On the contrary, the contacts needed to stabilize the acceptor in a favorable conformation to receive ^6^F-type products were likely weakened. A higher production of 1-kestose and neokestose was observed for equivalent variants R369A/K/S from *B*. *licheniformis* 8-37-0-1 levansucrase^20^.

The combination of mutations in variant 3E7 (R370Q/K373R/F419W) resulted in a greater accumulation of 1-kestose and a higher production of an unknown disaccharide, probably formed of two fructosyl units (Fig. 2B). 1-kestose was obtained with a final yield of 2.3% (relative to the substrate sucrose) in the case of this variant (Fig. 2B, Table 3), around 6.8-fold the yield of the wild-type levansucrase. Many levansucrases are known to produce 1-kestose. For instance, this tetrasaccharide is the main product of the levansucrase from *G*. *diazotrophicus* under some reaction conditions^28^, and 1-kestose is synthesized and used as an acceptor by the levansucrase from *B*. *subtilis* to produce 1,6-nystose that is further elongated via β2,6 linkages^29^. Analysis of time course reactions of *B*. *megaterium* levansucrase and its variants revealed 1-kestose as one of the main products at early reaction stages (Supplementary Information, Figs S7 and S8), specially for variants of group 4. However, none of the variants analyzed in this work was able to use 1-kestose efficiently as acceptor to generate other ^1^F-type products (Supplementary Information, Fig. S9). As mentioned above, a recent study conducted with the inulosucrase from *Lactobacillus reuteri* 121 proposed a different elongation pathway for ^1^F- and ^6^F-type FOS^30^. Due to the lack of structural information regarding this inulosucrase we used the structures of the inulosucrase from *L*. *johnsonii* that contain sucrose (PDB 2yfs) and 1-kestose (PDB 2yft) in the active site to retrieve information about the network of interactions needed to sustain polymerization of ^1^F-type products. Residue R542 in *L*. *johnsonii* inulosucrase (equivalent to R370), can interact with the glucosyl-moiety of sucrose at the +1 subsite and only with ^1^F-type-FOS at the +2 subsite, while R545 (K373 in *B*. *megaterium* levansucrase) can form hydrogen bonds with sucrose at the +1 subsite and with 1-kestose at the +1 and +2 subsites (Supplementary Information, Fig. S1). These contacts are not sufficient to support oligosaccharide elongation, and different carbohydrate interacting regions beyond the +2 subsite are proposed to be essential for binding and elongation of oligosaccharides containing β2,1 bonds in the main chain^30^. These regions are not found in the levansucrase from *B*. *megaterium*, probably being the reason why polymerization of ^1^F-type-FOS larger than 1-kestose was not catalyzed by the wild-type levansucrase or its variants (Supplementary Information, Fig. S1).

**Table 3:** Quantification of oligosaccharides produced by *B*. *megaterium* levansucrase variants from 0.5 m sucrose.

Variant	Position			1-kestose mm	6-Kestose mm	6-nystose mm	Unknown Disaccharide^a^	Neokestose^a^
	370	373	419					
WT	R	K	F	1.68 ± 0.27	0.35 ± 0.01	1.29 ± 0.14	1.00	1.00
1H11	**H**	K	F	4.99 ± 0.30	0.54 ± 0.06	1.10 ± 0.10	1.21 ± 0.07	0.88 ± 0.04
2B12	**H**	**S**	F	2.63 ± 0.19	3.36 ± 0.26	1.05 ± 0.10	0.95 ± 0.03	0.92 ± 0.05
1G2	R	**R**	F	3.57 ± 0.21	2.51 ± 0.17	4.68 ± 0.13	1.77 ± 0.12	2.04 ± 0.18
2H11	R	**R**	**Y**	4.67 ± 0.24	2.65 ± 0.12	5.17 ± 0.49	1.87 ± 0.03	1.47 ± 0.13
3H5	R	**R**	**W**	3.78 ± 0.17	1.91 ± 0.12	4.09 ± 0.35	1.95 ± 0.13	2.06 ± 0.15
2A5	**Q**	K	F	8.06 ± 0.39	0.68 ± 0.09	2.12 ± 0.16	2.86 ± 0.19	4.00 ± 0.19
5F11	**Q**	K	**W**	8.45 ± 0.34	0.91 ± 0.06	2.31 ± 0.15	3.14 ± 0.21	2.97 ± 0.10
3G5	**Q**	K	**Y**	9.70 ± 0.60	0.59 ± 0.05	2.00 ± 0.11	2.47 ± 0.18	2.93 ± 0.08
3E7	**Q**	**R**	**W**	11.50 ± 0.83	1.31 ± 0.08	1.62 ± 0.17	3.98 ± 0.13	2.72 ± 0.17
1B1	R	**H**	F	2.33 ± 0.17	12.94 ± 0.69	7.32 ± 0.46	1.92 ± 0.08	1.65 ± 0.02
2E11	R	**H**	**Y**	2.51 ± 0.12	11.62 ± 0.21	6.29 ± 0.17	1.75 ± 0.02	1.34 ± 0.10
3C5	R	**L**	F	2.27 ± 0.13	9.73 ± 0.35	5.49 ± 0.37	3.05 ± 0.03	3.42 ± 0.15
4E1	R	**L**	**Y**	2.21 ± 0.06	9.70 ± 0.18	4.34 ± 0.18	2.78 ± 0.03	2.47 ± 0.14

Reactions were performed in Sörensen buffer employing 2 U mL^−1^ of each enzyme. The products were analyzed by HPAEC-PAD after approximately 90% sucrose was converted (reached in a range of 20–28 h reaction for all variants). Mean values and standard deviations of three independent experiments are shown. Mutated residues are shown as underlined bold letters.

^a^For this sugar the peak relative area regarding to that of the wild type enzyme (set to 1.00) is provided.

The concentration of blastose and neokestose in reactions with *B*. *megaterium* wild-type levansucrase increases over the reaction course and neokestose is largely hydrolyzed to blastose once sucrose is depleted^18^. Group 4 variants hydrolyzed neokestose only partially at the final reaction stage (Supplementary Information, Fig. S8).

Due to the importance of R370 in coordinating glucose or fructose in the +1/+2 subsites, substitution of arginine by glutamine had a large effect on both K_M_ and k_cat_ values of enzymes in this group, as shown for variants 2A5 and 3E7 (R370Q, and R370Q/K373R/F419W) (Table 2).

All variants of group 5 contain the native arginine at position 370, and thus the capacity of these enzymes to synthesize ^6^F-type tri- and tetrasaccharides was not anticipated to be affected. The substitution of K373 by either histidine or leucine, however, hampered the capacity of these variants for further elongating 6-kestose, 6-nystose and an oligosaccharide that may correspond to levano-pentaose. Since these FOS could not be used efficiently as acceptors, their yields increased considerably in reactions employing variants of group 5. Variant 1B1 (K373H) for instance, produced 37- and 5.6-times more 6-kestose and 6-nystose, respectively, than the wild-type enzyme (Table 3, Fig. 2C). Mutations K373H and K373L had presumably a distinct effect in FOS binding than mutations K373A and K373R previously reported for *B*. *megaterium* and *B*. *licheniformis* levansucrases. In the former enzyme, mutation K373A reduced the formation of tri- and tetrasaccharides, slightly increasing the production of penta- and hexasaccharides, while mutation K373R enabled the variant to produce larger FOS of DP ≤ 8–9, probably by reinstalling some of the native oligosaccharide-enzyme interactions^19^. The crystal structure of variant K373A (PDB 3om4) showed that the substitution by alanine did not disturb the catalytic residues and had only a small effect on the conformation of the side chains of residues spatially close to the mutation^19^. The analogous mutation (K372A) in *B*. *licheniformis* levansucrase resulted in the synthesis of neokestose > 1- kestose > 6-kestose^20^. While arginine and histidine are expected to provide stabilization of growing oligosaccharides via hydrogen bonds, leucine is not. The product profile of mutants K373H and K373L was nonetheless similar (Supplementary Information, Fig. S10), indicating that both leucine and histidine were perhaps unable to interact with products larger than DP 6, which is needed to sustain polymerization of ^6^F-type FOS.

Variant K373H maintained a k_cat_ value similar to the wild-type levansucrase, although its K_M_ increased by around 4-fold. On the contrary, K_M_ of variant K373L was not affected, while its k_cat_ was reduced by 40% (Table 2).

### Synthesis and purification of tri- and tetrasaccharides

**Figure 3:**
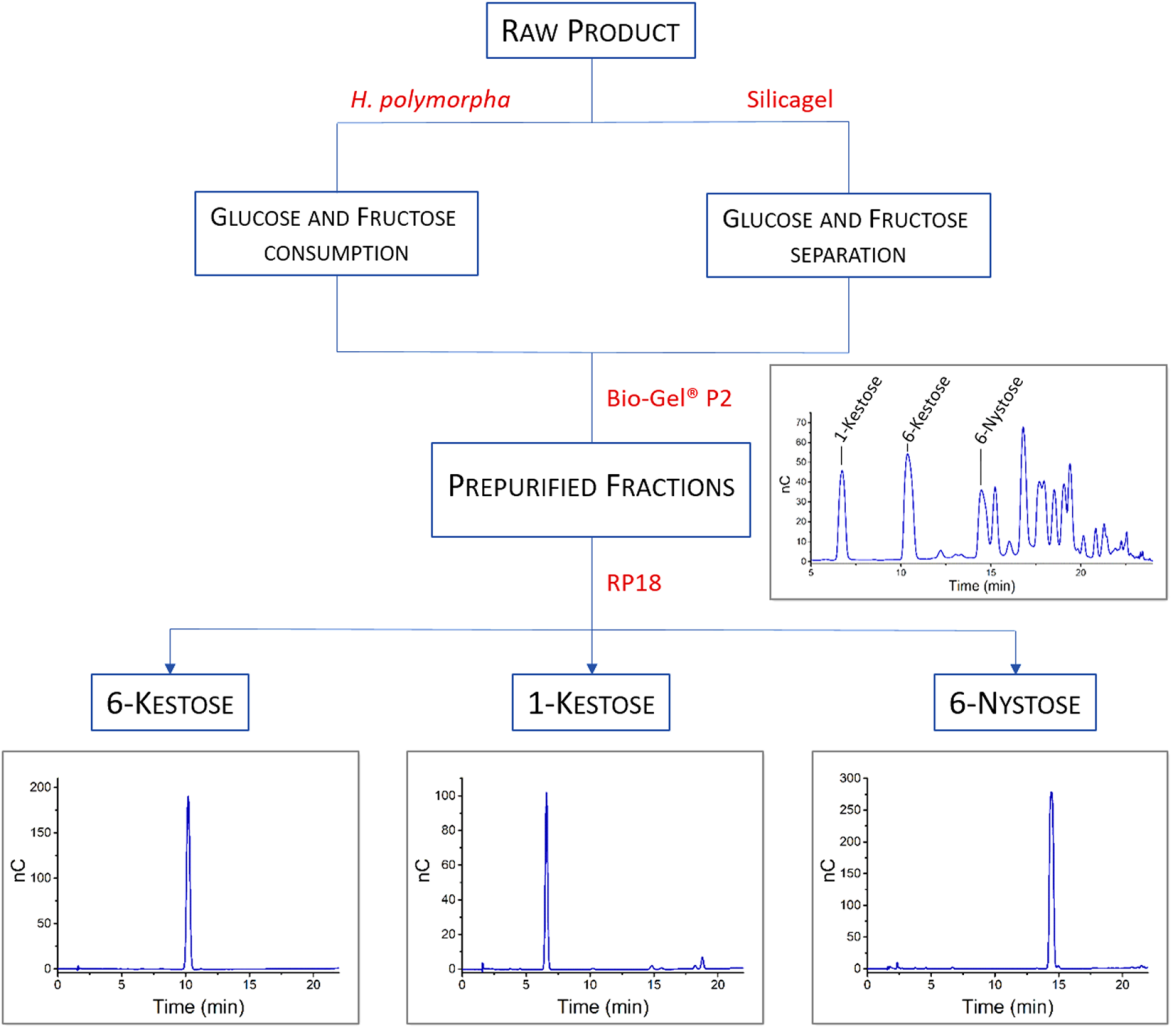
Purification process of oligosaccharides. Chromatogram depicting the outcome of the Bio-Gel® P2 column corresponds to one of the 10 pooled fractions that were used for further purification through the RP18 resin. The amount of specific FOS relative to the other products varies in each fraction. Uncropped versions of these chromatograms are shown as Supplementary Information, Fig. S12.

Variant 3C5 (K373L), which displayed the best catalytic efficiency among enzymes from group 5 was selected for the synthesis and isolation of 1-kestose, 6-kestose and 6-nystose, with special focus on the last two products. Glucose, fructose and residual sucrose are the main sub-products in oligosaccharides synthesis, often hampering the downstream processing. Frequent methods employed in fractionation and purification of FOS include ultra- and nano-filtration^31^. Microbial treatment with organisms that are unable to degrade the target products while consuming glucose, fructose and residual sucrose to generate ethanol and carbon dioxide is another method of choice for facilitating the purification process^31,32^. Two strategies were followed in this work for the small-scale production and purification of the aforementioned oligosaccharides: 1) column chromatography and 2) a combination of a fermentative treatment of the crude oligosaccharide mixture with the yeast *Hansenula polymorpha* and column chromatography (Fig. 3 and Supplementary Information, Fig. S11). *H*. *polymorpha* was expected to be a suitable yeast for purification of the FOS mixture, since it contains a sucrose hydrolyzing maltase, but no FOS degrading invertases^33^.

A mixture of FOS with residual glucose and fructose was obtained after incubation of 125 mL of a solution containing 0.5 m sucrose and 3 U mL^−1^ of variant K373L at 37 °C for 48 h. After this reaction time there were only traces of sucrose left, which facilitated the purification procedure and limited the first separation step to the monosaccharides. The concentrations of produced glucose and fructose in the raw product were 248 mm and 194 mm, respectively. Both fermentative and chromatographic separation of glucose and fructose were performed in quantitative yields and without noteworthy loss of FOS (Supplementary Information, Figs S11 and S12). However, chromatographic purification is accompanied by a much higher outlay including the time input as well as economic and environmental aspects. Particularly for large-scale applications, a high quantity of organic solvents and unrecyclable silica gel would be required. Glycerol byproducts emerging during the fermentation process were easily separated in subsequent purification stages, which include the use of Bio-Gel^®^ P2 and RP18 resins. Starting with 19.5 g of the raw product (Fig. 3) containing 155 mg 1-kestose, 197 mg 6-kestose and 316 mg 6-Nystose, we were able to isolate 1-kestose (43 mg), 6-kestose (111 mg) and 6-nystose (62 mg), which corresponds to 28, 56 and 20% of those substances contained in the crude FOS mixture.

All products were characterized by mass spectroscopy and NMR (Supplementary Information, Figs S13–S18).

## Conclusions

The association of the gut microbiome composition with the occurrence and development of both gastrointestinal and extragastrointestinal diseases in humans has been in the spotlight over the last decades. Special attention has been devoted to the bidirectional communication between the central and enteric nervous systems, the so-called “gut-brain-axis”^1^, with several studies analyzing the manner in which the gut microbiome influences such interactions^34^. Because molecules with prebiotic activity strongly effect the composition and number of colonic microbial populations, inclusion of prebiotics in dairy and other food formulations is gaining momentum. While the function of readily available inulin-type oligosaccharides has long been studied, there are fewer reports involving ^6^F-molecules either as a mixture or as purified single oligosaccharides. In part, this is justified by difficulties associated to the isolation of this type of sugars in sufficient yields to support studies. With few exceptions^35,36^, reported levansucrases often produce wide FOS distributions^16^ or polymers of several kDa^10^. Since 6-kestose and other small molecules are the acceptor substrates in the formation of large levan polymers, these sugars are usually not accumulated over the reaction course. We reported here the construction of a library of the levansucrase from *B*. *megaterium*, which was focused on the synthesis of short-chain oligosaccharides. Residues known for their role in modulating the size of ^1^F- and ^6^F-products were revisited to analyze the contribution of diverse amino acid side-chains to product specificity. In general variants containing the mutation R370Q produced preferentially 1-kestose and neokestose, while variants carrying the mutation K373H or K373L generate 6-kestose and 6-nystose as the main products. Based on a combination of microbial fermentation of side-products and chromatographic steps molecules 1-kestose, 6-kestose and 6-nystose were isolated with a purity superior to 95% (based on HPAEC-PAD analysis). Although the strategy for production and purification of these sugars was not optimized in this work and requires further development, it proved how engineered levansucrases can be applied for gaining access to custom oligosaccharides.

## Methods

### Multiple Sequence Alignment of Glycoside hydrolase family 68

One thousand protein sequences from family GH68 were extracted from UniProt using *B*. *megaterium* levansucrase as a query (D5DC07). Proteins with a similarity threshold of 0.95 were clustered with CD-Hit^37^ to reduced sequence redundancy. Sequences not containing the conserved GH68 sequence motifs ^94^WD^95^, ^172^WXGX^175^, ^256^RDP^258^, ^349^DXXER^353^, and ^421^YSX^423^ which are part of the central cavity of the β-propeller, were also discarded. Non-redundant sequences (372) were aligned with MUSCLE^38^ (Supplementary Information, Table S1) and amino acid distribution for positions 370, 373 and 419 were analyzed.

### Creation of a recombination levansucrase library at positions R370, K373 and F419

The library was generated following the method described previously^16^. In brief, three PCR fragments were generated which contained codon exchanges at position R370, K373 and F419. For each of the desired codon exchange an individual oligonucleotide was ordered and separate PCRs were performed. The resulting PCR fragments were mixed, recombined to the full levansucrase gene and cloned into the expression vector (derivative of pRSF, Novagen). *E*. *coli* BL21(DE3) was transformed for expression of the library variants. Inserts from 15 random selected clones were sequenced to validate the library quality. No unwanted mutation and an even distribution of the desired codons were obtained.

### Expression of the levansucrase library in deep 96-well plates

Single clones from agar plates containing 10 mg mL^−1^ kanamycin were used to inoculate 400 µL of ZYM505/well containing the appropriate antibiotic. The plates were covered with an air permeable foil and grown at 37 °C overnight in a TIMIX incubator at 1000 rpm. The final OD_600_ for each well was around 10–15. These pre-cultures were used to inoculate 1 mL ZYM505/well with appropriate antibiotic (starting OD_600_ around 0.05). Plates were shaken at 37 °C until an OD600 of 0.6–1.0 was reached. Expression was induced by adding IPTG at a final concentration of 0.5 mm. Cultures were incubated overnight at 20 °C. Cells were harvested by centrifugation and resuspended in 100 µL lysis-buffer/well (50 mm Tris pH 7.0; 2 mm MgCl2; 1x Cellytic B; 0.1 mg mL^−1^ lysozyme and 20 U mL^−1^  benzonase). Deep well plates were incubated at 30 °C for 45 min in a TIMIX incubator at 1000 rpm and afterwards they were centrifuged at 3000 × g for 30 min. Cleared cell lysates were transferred to 96-well microtiter plates and used directly for screening assays.

### Screening for initial activity in 96-well microtiter plates

Enzyme extracts were used to determine the initial activity at 37 °C by adding 1 µL crude extract to 99 µL sucrose solution (0.5 M sucrose and 50 mm Sörensen buffer pH 6.6) in 96 well plates. Sörensen buffer contains 50 mm Na_2_HPO_4_ and 50 mm KH_2_PO_4_. The reactions were stopped after 10 min by adding 100 µL DNS reagent. Plates were heated at 95 °C for 5 min and cooled down for 2 min at 4 °C. For quantification, samples were diluted 1:6 with water and the absorbance measured at 540 nm. Data was processed using a calibration curve of absorbance versus glucose concentration.

### Analysis of product profiles on TLC

Enzyme extracts were used to determine the product profile of *B*. *megaterium* levansucrase variants on TLC. Reactions were performed in 96-well plates containing 1 µL enzyme crude extract and 99 µL sucrose solution (0.5 M sucrose and 50 mm Sörensen buffer pH 6.6). Plates were incubated at 37 °C during 20 h and reactions stopped with 100 µL of 50 mm NaOH. Standards and 1 µL of each enzymatic reaction were applied on TLC plates (silica gel 60 F_254_ with concentration zone), dried and run three times using WIE (water/iso-propanol/ethyl acetate 1/3/6) as eluent. The plates were dried and stained with *N*-(1-naphtyl)-ethylenediamine (0.3% m/v) in 5.0% (v/v) conc. H_2_SO_4_ and 94.7% (v/v) methanol.

### Expression and purification of levansucrase variants

One single colony of freshly transformed *E*. *coli* bearing selected plasmids was used to inoculate 10 mL LB-medium containing 10 µg mL^−1^ kanamycin. Precultures were incubated over night at 37 °C and used to inoculate 250 mL LB-medium with appropriate antibiotic. Expression of levansucrase was induced when cells reached an OD_600_ of around 0.6 by adding IPTG at a final concentration of 0.5 mm. Cultures were incubated over night at 20 °C. Cells were harvested by centrifugation and resuspended in 7 mL of 20 mm Sörensen buffer pH 6.0. After sonication, extracts were cleared by centrifugation at 13,000 × g. Cleared lysates were loaded onto a CM sepharose column and levansucrase eluted with a linear gradient of Sörensen buffer from 20 mm to 1.0 m. Purification fractions were pooled and concentrated. An extinction coefficient of 64750 M^−1^ cm^−1^ and a path length of 1 cm were used to calculate concentration of purified enzymes measured at 280 nm.

### Analysis and quantification of oligosaccharides by HPAEC-PAD

Enzyme extracts were used to determine the product profile of *B*. *megaterium* levansucrase variants. 1 mL reactions containing 50 mm Sörensen buffer pH 6.6, 0.5 mm sucrose and 2 U mL^−1^ of each enzyme were incubated at 37 °C. Samples (50 µL) were taken over the reaction course and enzymes inactivated by adding an equal volume of 50 mm NaOH. After centrifugation (16,000 × g) to remove precipitated protein the samples were diluted 1,000-times and analyzed by HPAEC-PAC. Analysis of oligosaccharides was performed with a Dionex ICS-5000+ SP system utilizing a CarboPac PA10 column with pulsed amperometric detection. Elution solvents employed for separation were 100 mm NaOH (A) and 100 mm NaOH/1 M NaOAc (B). A multistep gradient was programmed as follows: 0–5 min 100% A, 5–30 min 0–50% B, 30–45 min 100% A. Appropriate standards were used for quantification of some products, while comparison of relative areas was utilized for products with unknown structures. The concentration (or peak area) of oligosaccharide products was calculated from three independent experiments.

### Determination of kinetic parameters

Catalytic parameters of levansucrase variants were obtained from reactions carried out in 50 mm Sörensen buffer pH 6.5 containing varying concentrations of sucrose and purified levansucrase variants. Initial rates were determined by the DNS method, quantifying the release of reducing sugars over the time course. K_M_ and k_cat_ values were calculated by fitting the data to the Michaelis-Menten equation using a non-linear curve in OriginPro (OriginLab). Experiments were performed in duplicate.

### Synthesis and purification of oligosaccharides produced by variant K373L

125 mL of an aqueous solution containing 0.5 m sucrose, 50 mm Sörensen buffer pH 6.6 and 3 U mL^−1^ of the levansucrase variant K373L were incubated at 37 °C for 48 h. The solution was stopped by heating at 95 °C for 10 minutes. After filtration of the mixture (0.2 µm exclusion size) the solvent was removed under reduced pressure to obtain 19.5 g of a colorless crude product containing mono-, di- and oligosaccharides.

For the elimination of the high glucose and fructose content two methods were tested: normal phase column chromatography with silica gel and fermentation with the yeast *H*. *polymorpha*. The eluent for the silica gel column was H_2_O/isopropanol/ethyl acetate (1:3:6). For the fermentation of glucose and fructose, 5 g of the crude product were dissolved in 150 mL ddH_2_O and 50 g of immobilized *H*. *polymorpha* yeast were added to consume the monosaccharides. The mixture was incubated at 25 °C for 24 h. The fermentation reaction was inactivated by heating at 95 °C for 10 min. Yeast alginates were withdrawn by filtration and the solvent was removed under reduced pressure to yield 2.9 g of a colorless syrup. The syrup was purified over the size exclusion column Bio-Gel® P2 (eluent ddH_2_O) to obtain FOS with a degree of polymerization of 3–4 and impurities of glycerol. Further purification with reversed phase chromatography (RP-18, eluent ddH_2_O) led to the desired products 1-kestose (43 mg, 28% of 1-kestose content in 19.5 g crude product), 6-kestose (111 mg, 56%) and 6-nystose (62 mg, 20%).

### Cultivation of *H*. *polymorpha*

The pre-cultures of 5 mL YM-medium were inoculated by selecting one single colony of *H*. *polymorpha*. After incubation overnight at 25 °C the pre-cultures were used for inoculation of 250 mL YM-medium. The cells of the main culture were grown at 25 °C and 140 rpm for 24 h. The yeast cells were harvested by centrifugation at 8,000 rpm for 10 min. The supernatant was discarded and the cells were immediately used for immobilization or stored at −20 °C.

YM-medium contains yeast extract (3.0 g), malt extract (3.0 g), peptone from soybeans (5.0 g) and glucose (10.0 g). It was filled up with distilled water to 1 L.

### Immobilization of *H*. *polymorpha*

1.56 g alginate was added to 100 mL ddH_2_O and heated to 70 °C until a viscous solution resulted. After cooling the alginate solution to room temperature 12.5 g of *H*. *polymorpha* (wet weight) was added to give a homogeneous suspension. The alginate-yeast suspension was dropped steadily with a syringe pump to a 150 mm calcium chloride solution. After the immobilized beads were stirred for 1 h to harden, they were filtrated and used immediately or within the next 24 h.

### Characterization of products by NMR

#### 1-Kestose

^**1**^**H-NMR** (600 MHz, D_2_O): σ = 5.40 (1H, d, ^3^*J* = 3.8 Hz, *H*_Glu_-1), 4.25 (1H, d, ^3^*J* = 8.7 Hz, *H*_ru1_-3′), 4.16 (1H, d, ^3^*J* = 8.5 Hz, *H*_Fru2_-3″), 4.05 (1H, dd, ^3^*J* = 8.5, 8.5 Hz, *H*_Fru2_-4″), 4.02 (1H, dd, ^3^*J* = 8.7, 8.7 Hz, *H*_Fru1_-4′), 3.86–3.83 (2H, m, *H*_Fru1_-5′, *H*_Fru2_-5″), 3.86 – 3.79 (1H, m, H_Fru2_-1″a), 3.83–3.80 (2H, m, *H*_Fru1_–6′b, *H*_Glu_-5), 3.83–3.74 (4H, m, *H*_Fru1_-6′, *H*_Fru2_-6″), 3.82–3.78 (1H, m, *H*_Fru1_-1′a), 3.79–3.77 (2H, m, *H*_Glu_-6), 3.74–3.71 (1H, dd, ^3^*J* = 9.9, 9.6 Hz, *H*_Glu_-3), 3.73–3.64 (1H, m, *H*_Fru2_-1″b), 3.71–3.67 (1H, m, *H*_Fru1_-1′b), 3.52 (1H, dd, ^3^*J* = 9.9, 3.8 Hz, *H*_Glu_-2), 3.46–3.43 (1H, dd, ^3^*J* = 9.6, 9.6 Hz, *H*_Glu_-4) ppm.

^**13**^**C-NMR** (150 MHz, D_2_O): σ = 103.7 (*C*_Fru2_-2″), 103.2 (*C*_Fru1_-2′), 92.4 (*C*H_Glu_-1), 81.1 (2C, *C*H_Fru1_-5′, *C*H_Fru2_-5″), 76.5 (2C, *C*H_Fru1_-3′, *C*H_Fru2_-3″), 74.4 (*C*H_Fru2_-4″), 73.8 (*C*H_Fru1_-4′), 72.5 (*C*H_Glu_-3), 72.3 (*C*H_Glu_-5), 71.1 (*C*H_Glu_-2), 69.1 (*C*H_Glu_-4), 62.2, (2C, *C*H_2_,_Fru1_-6′, *C*H_2_,_Fru2_-6″), 60.8 (2C, *C*H_2_,_Fru1_-1′, *C*H_2_,_Fru2_-1″), 60.0 (*C*H_2,Glu_-6) ppm.

#### 6-Kestose

^**1**^**H-NMR** (600 MHz, D_2_O): σ = 5.35 (1H, d, ^3^*J* = 3.9 Hz, *H*_Glu_-1), 4.17 (1H, d, ^3^*J* = 8.7 Hz, *H*_Fru1_-3′), 4.14 (1H, d, ^3^*J* = 8.4 Hz, *H*_Fru2_-3″), 4.07 (1H, dd, ^3^*J* = 8.4, 8.0 Hz, *H*_Fru2_-4″), 4.02 (1H, dd, ^3^*J* = 8.7, 8.6 Hz, *H*_Fru1_-4′), 3.94 – 3.91 (2H, m, *H*_Fru1_-5′, *H*_Fru1_-6′a), 3.86–3.84 (1H, m, H_Fru2_-5″), 3.84–3.79 (2H, m, *H*_Fru1_-6′b, *H*_Fru2_-6″a), 3.83–3.77 (3H, m, *H*_Glu_-6a, *H*_Glu_-6b, *H*_Glu_-5), 3.77–3.75 (1H, m, *H*_Fru2_-1″a), 3.74–3.71 (1H, dd, ^3^*J* = 9.8, 9.5 Hz, *H*_Glu_-3), 3.66–3.59 (4H, m, *H*_Fru1_-1′, *H*_Fru2_-1″b, *H*_Fru2_-6″b), 3.54–3.52 (1H, dd, ^3^*J* = 9.8, 3.9 Hz, *H*_Glu_-2), 3.45–3.42 (1H, dd, ^3^*J* = 9.5, 9.5 Hz, *H*_Glu_-4) ppm.

^**13**^**C-NMR** (150 MHz, D_2_O): σ = 103.7 (2C, *C*_Fru1_-2′, *C*_Fru2_-2″), 92.1 (*C*H_Glu_-1), 81.1 (*C*H_Fru2_-5″), 80.3 (*C*H_Fru1_-5′), 76.4 (*C*H_Fru2_-3″), 76.0 (*C*H_Fru1_-3′), 74.6 (*C*H_Fru2_-4″), 74.3 (*C*H_Fru1_-4′), 72.5 (*C*H_Glu_-3), 72.4 (*C*H_Glu_-5), 71.0 (*C*H_Glu_-2), 69.2 (*C*H_Glu_-4), 62.9 (*C*H_2_,_Fru1_-6′), 62.6 (*C*H_2_,_Fru2_-6″), 61.1 (*C*H_2_,_Fru1_-1′), 60.2 (*C*H_2_,_Glu_-6), 59.7 (*C*H_2_,_Fru2_-1″) ppm.

#### 6-Nystose

^**1**^**H-NMR** (600 MHz, D_2_O): σ = 5.40 (1H, d, ^3^*J* = 3.9 Hz, *H*_Glu_-1), 4.24 (1H, d, ^3^*J* = 8.6 Hz, *H*_Fru1_-3′), 4.16 (1H, d, ^3^*J* = 8.5 Hz, *H*_Fru3_-3″′), 4.15 (1H, d, ^3^*J* = 8.5 Hz, *H*_Fru2_-3″), 4.10 (1H, dd, ^3^*J* = 8.5, 8.5 Hz, *H*_Fru2_-4″), 4.04 (1H, dd, ^3^*J* = 8.6, 8.6 Hz, *H*_Fru1_-4′), 4.02 (1H, dd, ^3^*J* = 8.5, 7.8 Hz, *H*_Fru3_-4″′), 3.94–3.91 (2H, m, *H*_Fru3_-5″′, *H*_Fru3_-6″′a), 3.87–3.84 (2H, m, *H*_Fru1_-5′, *H*_Fru2_-5″), 3.83–3.82 (1H, m, *H*_Fru2_-1″a), 3.83–3.76 (3H, m, *H*_Fru1_-6′, *H*_Fru2_-6″a), 3.82–3.80 (1H, m, *H*_Glu_-5), 3.82–3.78 (2H, m, *H*_Glu_-6), 3.81 (2H, m, *H*_Fru3_-1″′), 3 0.76–3.75 (1H, m, *H*_Fru1_-1′a), 3.75–3.72 (1H, m, *H*_Fru3_-6″′b), 3.73 (1H, dd, ^3^*J* = 9.6, 9.6 Hz, *H*_Glu_-3), 3.68–3.64 (1H, m, *H*_Fru2_-6″b), 3.67–3.65 (2H, m, *H*_Fru2_-1″b), 3.52 (1H, dd, ^3^*J* = 9.6, 3.9 Hz, *H*_Glu_-2), 3.45 (1H, dd, ^3^*J* = 9.6, 9.6 Hz, *H*_Glu_-4) ppm.

^**13**^**C-NMR** (150 MHz, D_2_O): σ = 103.9 (*C*_Fru3_-2″′), 103.7 (*C*_Fru2_-2″), 103.2 (*C*_Fru1_-2′), 92.4 (*C*H_Glu_-1), 81.2 (*C*H_Fru2_-5″), 81.1 (*C*H_Fru1_-5′), 80.2 (*C*H_Fru3_-5″′), 76.5 (*C*H_Fru2_-3″), 76.4 (*C*H_Fru1_-3′), 76.3 (*C*H_Fru3_-3″′), 75.1 (*C*H_Fru3_-4″′), 74.6 (*C*H_Fru2_-4″), 73.7 (*C*H_Fru1_-4′), 72.5 (*C*H_Glu_-3), 72.3 (*C*H_Glu_-5), 71.0 (*C*H_Glu_-2), 69.1 (*C*H_Glu_-4), 63.1 (*C*H_2_,_Fru3_-6″′), 62.5 (*C*H_2_,_Fru2_-6″), 62.0 (*C*H_2_,_Fru1_-6′), 60.7 (*C*H_2_,_Fru1_-1′), 60.3 (*C*H_2,Glu_-6), 60.0 (*C*H_2_,_Fru3_-1″′), 59.8 (*C*H_2,Fru2_-1″) ppm.

## Supplementary information

Supplementary Information

## Data Availability

All data generated or analyzed during this study are included in this published article (and its Supplementary Information files).
