# Supplementary material for: Exploring the sequence variability of polymerization-involved residues in the production of levan- and inulin-type fructooligosaccharides with a levansucrase
Source: Sci Rep. 2019 May 22;9:7720. doi: 10.1038/s41598-019-44211-5 (PMC6531494; doi:10.1038/s41598-019-44211-5)
Supplement: Supplementary file 1 — Supplementary Information [file 41598_2019_44211_MOESM1_ESM.pdf]

Exploring the sequence variability of polymerization-involved residues in the production of levan- and inulin-type fructooligosaccharides with a levansucrase

Christian Possiel[1,3], Maria Elena Ortiz-Soto[1,3], Julia Ertl[1], Angela Münch[1], Andreas Vogel[2], Ramona Schmiedel[2], Jürgen Seibel[1*]

[1]Institute of Organic Chemistry, University of Würzburg, Würzburg, Am Hubland 97074, Germany.

[2]C-LEcta GmbH, Leipzig, Perlickstr. 5, 04103, Germany.

*e-mail: seibel@chemie.uni-wuerzburg.de

[3] These authors contributed equally to this work.

Supplementary Table S1. Amino acid distribution of the mutated positions in a multiple sequence alignment of the Glycoside Hydrolase Family 68 (372 non-redundant sequences). Sequences were aligned with MUSCLE. Values are given in percentage.

Position	Arg (R)	Asn (N)	Asp (D)	Gln (Q)	Glu (E)	Lys (K)	His (H)	Thr (T)	Phe (F)	Trp (W)	Tyr (Y)	Val (V)	Other
**370**	42.2	-	-	-	-	-	57.8	-	-	-	-	-	-
**373**	3.0	1.6	0.5	3.0	-	34.1	0.3	51.9	-	-	-	0.8	4.8
**419**	-	0.5	1.3	54.3	2.2	-	0.3	-	9.9	15.6	0.3	-	15.6

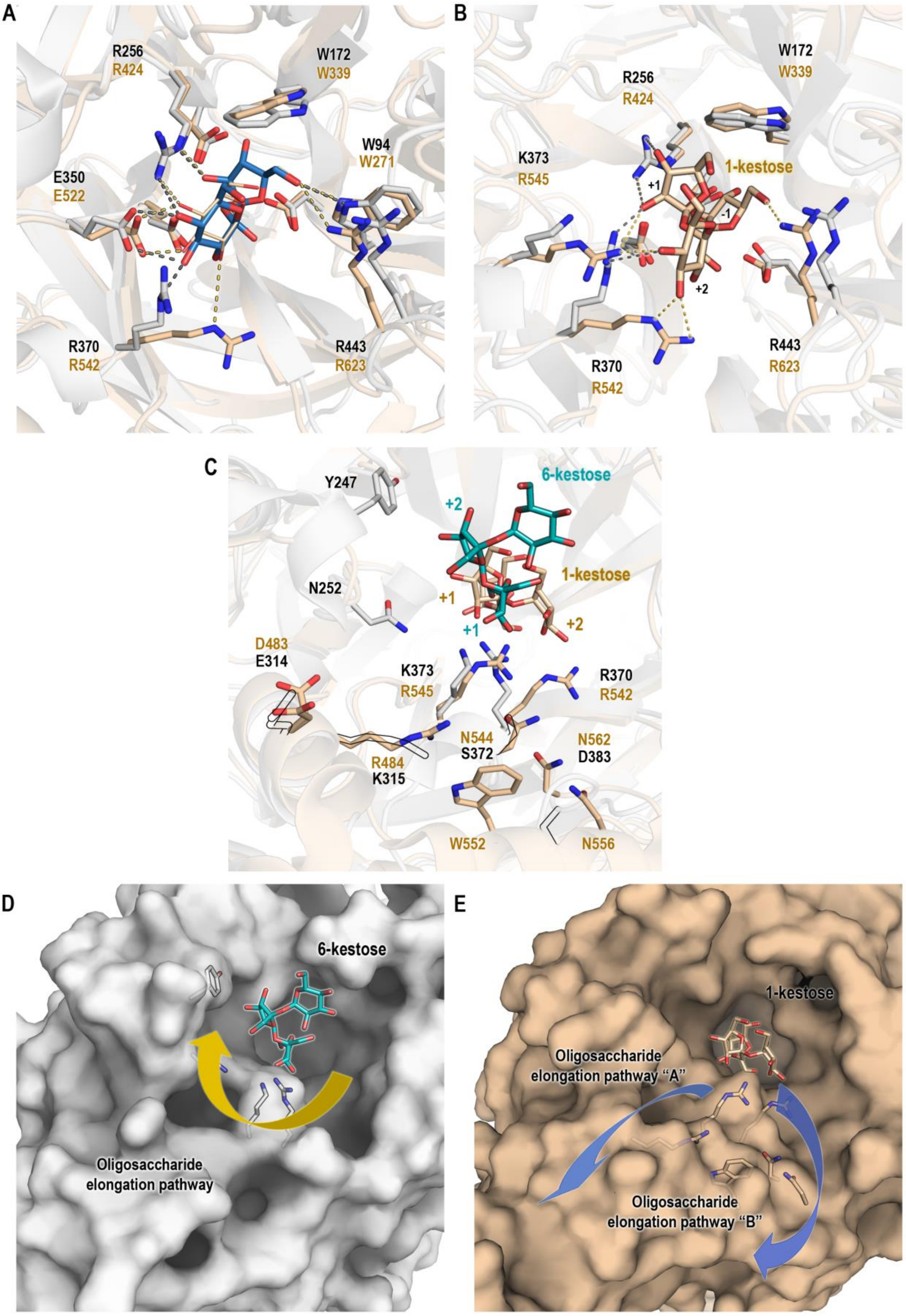

Supplementary Figure S1. Residues proposed to be in contact with donor and acceptor molecules in the levansucrase from *B. megaterium* (PDB 3om2/white) and the inulosucrase from *Lactobacillus*

reuteri. Due to the lack of structural information regarding this inulosucrase, some of the positions proposed to be in contact with [1]F-type FOS are shown in the inulosucrase from *L. johnsonii* (PDB 2yft/ochre). A) Residues that can form hydrogen bonds with the donor molecule at the -1 and +1 subsites (sucrose binding site). For clarity contacts with the catalytic amino acids are not displayed. Sucrose depicted in blue was aligned from the crystal structure of *B. subtilis* levansucrase (PDB 1pt2), while sucrose shown in ochre belongs to the crystal structure of *L. johnsonii* inulosucrase (PDB 2yfs). B) Residues at hydrogen bond distances to 1-kestose (from PDB 2yft). C) Residues defining the potential elongation pathways for inulin- and levan-type products[1-4]. 1- and 6-kestose are shown in the central binding pocket. 6-kestose from the 6-SST/6-SFT enzyme from *Pachysandra terminalis* (PDB 3ugh) is depicted in cyan. While 1-kestose occupies the -1, +1 and +2 subsites in *L. johnsonii* inulosucrase, the terminal fructose of 6-kestose binds to the +1, +2 and +3 subsites of *Pachysandra terminalis* fructosyltransferase. Due to structural differences between inulin- and levan-type oligosaccharides, the acceptor sugar moieties corresponding to the +2 position have a different orientation. Residues of *B. megaterium* levansucrase that are found at equivalent positions to those of the inulosucrase in the elongation pathway are shown only as silhouettes. D-E) Surface representation of the levansucrase from *B. megaterium* and inulosucrase from *L. johnsonii* with 6- and 1-kestose, respectively. The suggested routes for product growth[1,4] are indicated with arrows. Residues involved in saccharide-enzyme contacts are also depicted. Structural alignments were performed with UCSF Chimera [5].

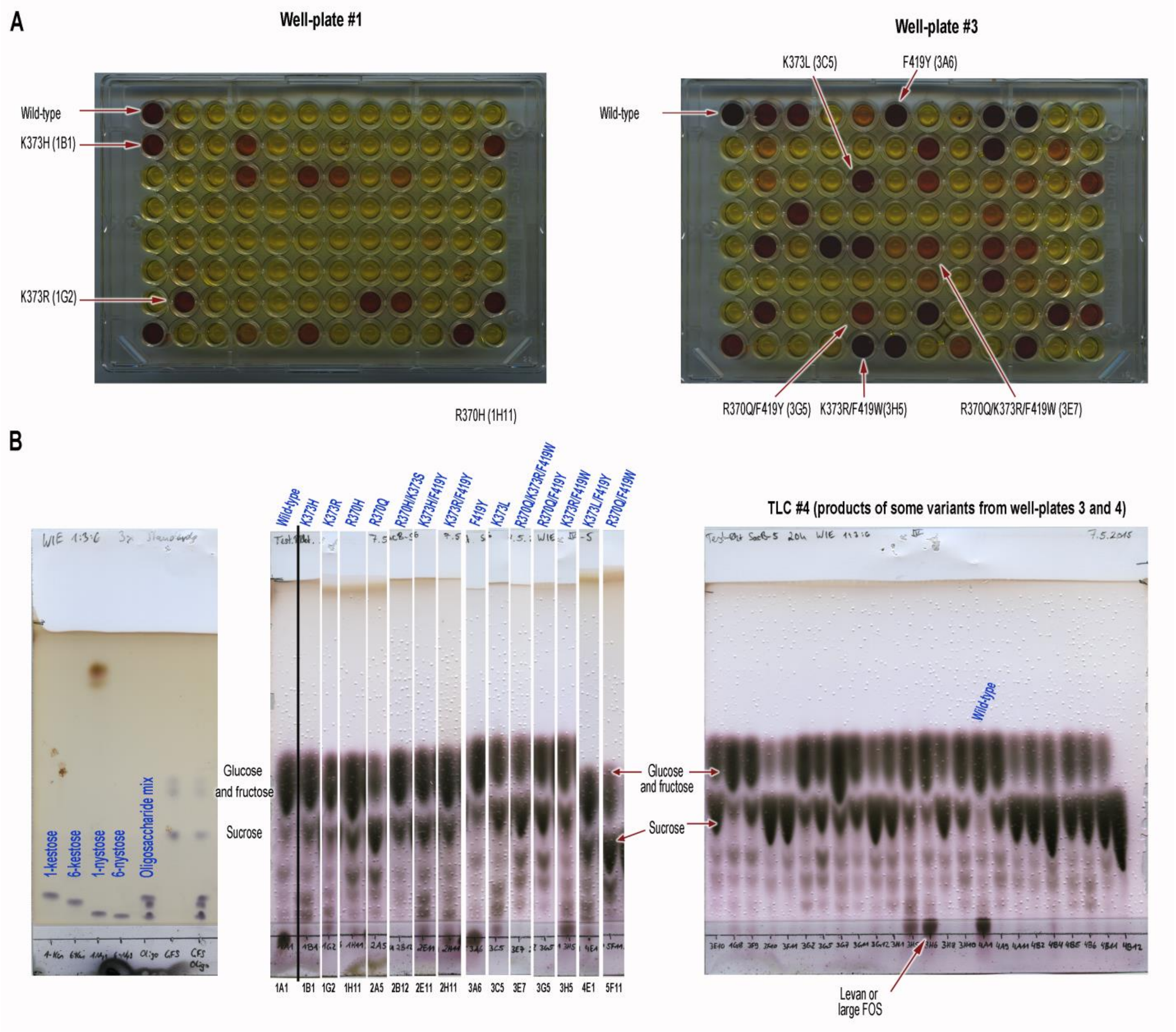

Supplementary Figure S2. Screening of *B. megaterium* levansucrase variants on 96-well plates (A) and Thin-layer chromatography (TLC) (B). 768 clones were assayed.

A) 96-well plates showing the activity assay (hydrolysis and transfer) of some variants. A dark orange-brown color indicates a higher release of reducing sugars and thus a higher global activity.

B) TLC plates containing standard sugars fructose, glucose, sucrose, 1-kestose, 6-kestose, 1-nystose and 6-nystose (left). Characterized variants mentioned in the main document are displayed (center, cropped lines from non-contiguous positions or from a different TLC plate.). The TLC plate at the right side is a representative of the screening method.

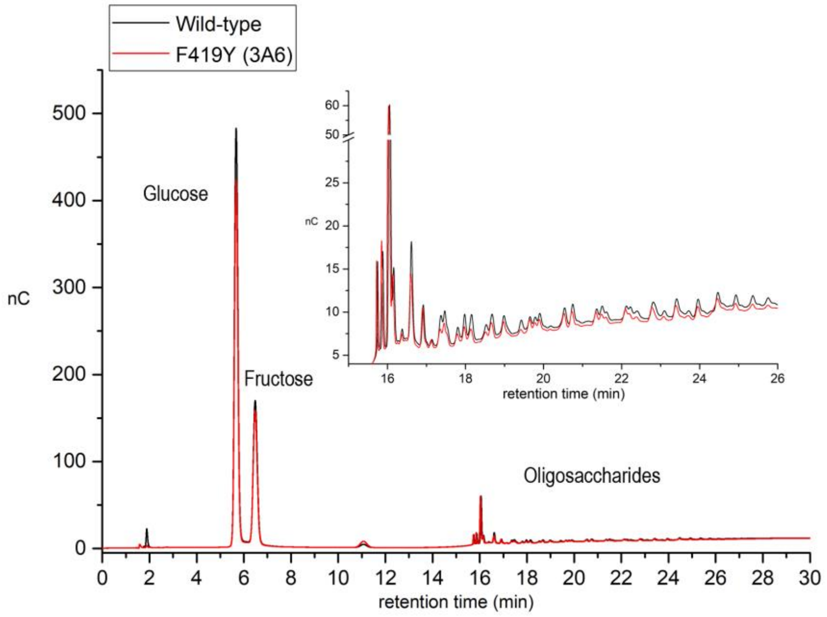

Supplementary Figure S3. Comparison of the HPAEC-PAC products of the wild-type levansucrase and variant F419Y (3A6).

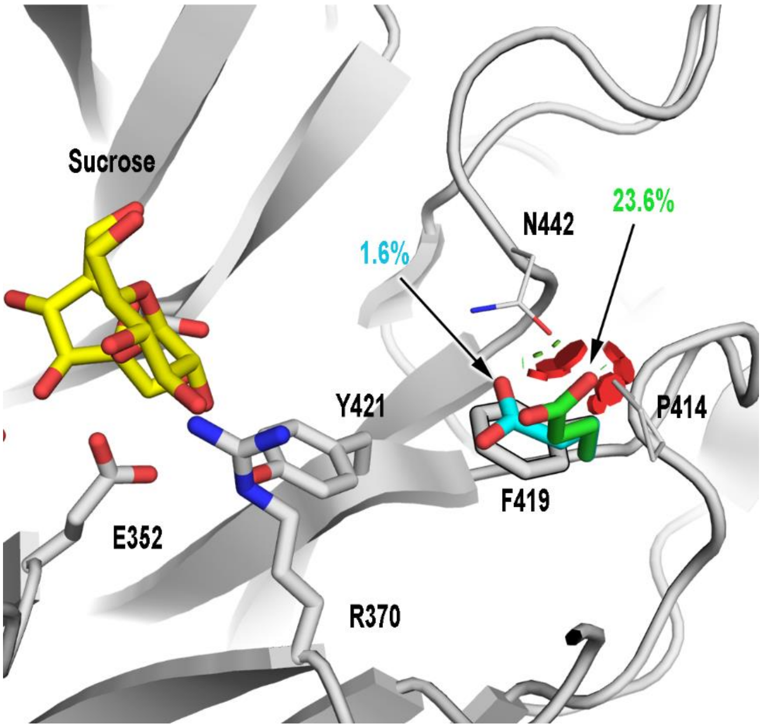

Supplementary Figure S4. Possible rotamers of the glutamic acid side chain for the mutation F419E. Rotamers (green and cyan) were obtained using the Mutagenesis Wizard function included in PyMol. The percentage of structures with a particular rotamer out of a sampled population is provided. Red polygons show probable clashes of a given rotamer with other residues in its vicinity. The carboxylate of the glutamic acid rotamer in green is at 1.9 Å from the carboxamide of N442, and at 2.1 Å of the P414 main chain. Only the rotamers for the mutation Y419E are shown; however, variant 3B9 also contains the mutation K373H.

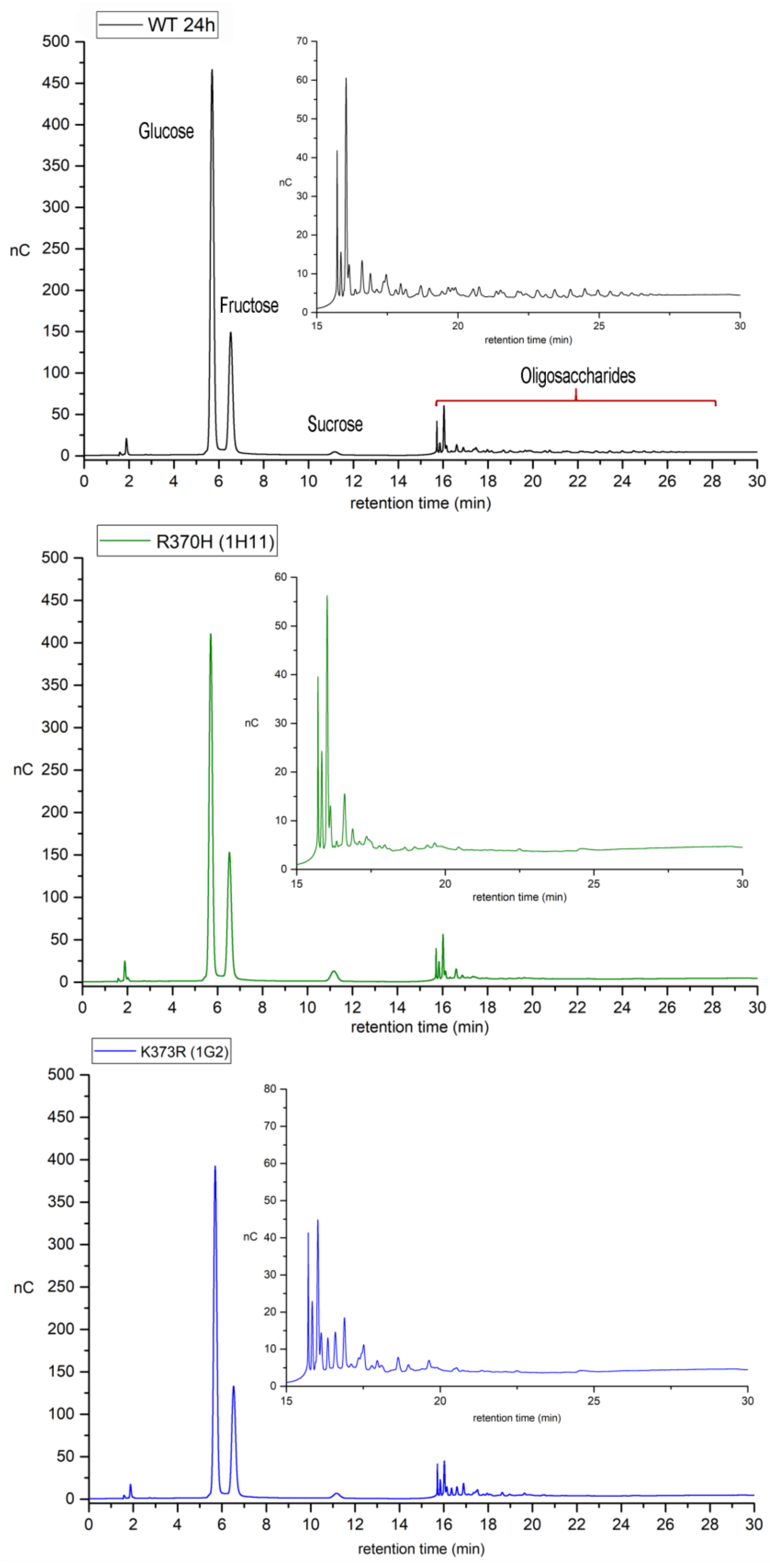

Supplementary Figure S5. Full-length HPAEC-PAC chromatograms showing the product profile of the wild-type levansucrase and variants 1H11 and 1G2. The cropped version of these chromatograms is shown in the main document (Figure 2).

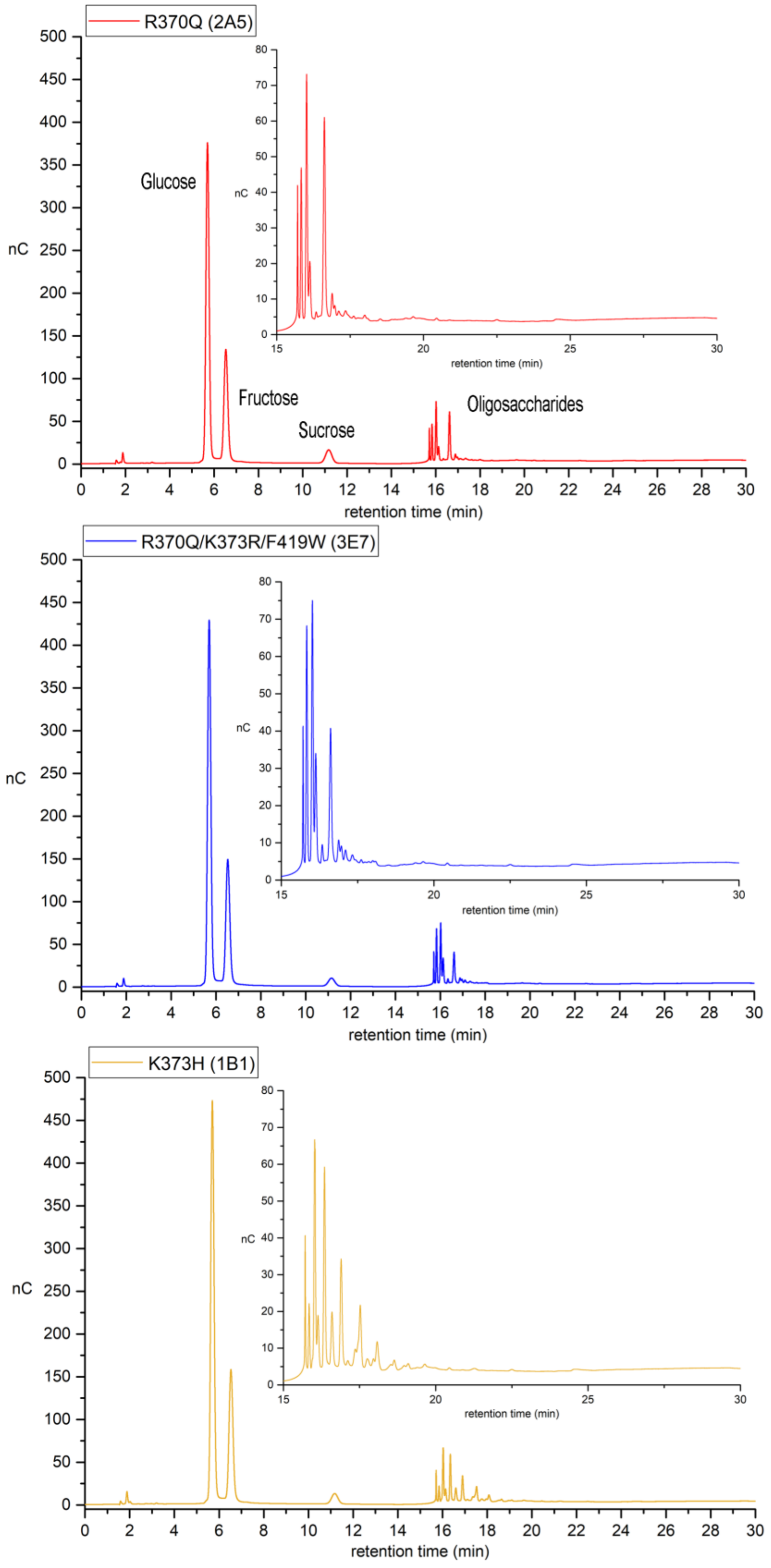

Supplementary Figure S6. Full-length HPAEC-PAC chromatograms showing the product profile of variants 2A5, 3E7 and 1B1. The cropped version of these chromatograms is shown in the main document (Figure 2).

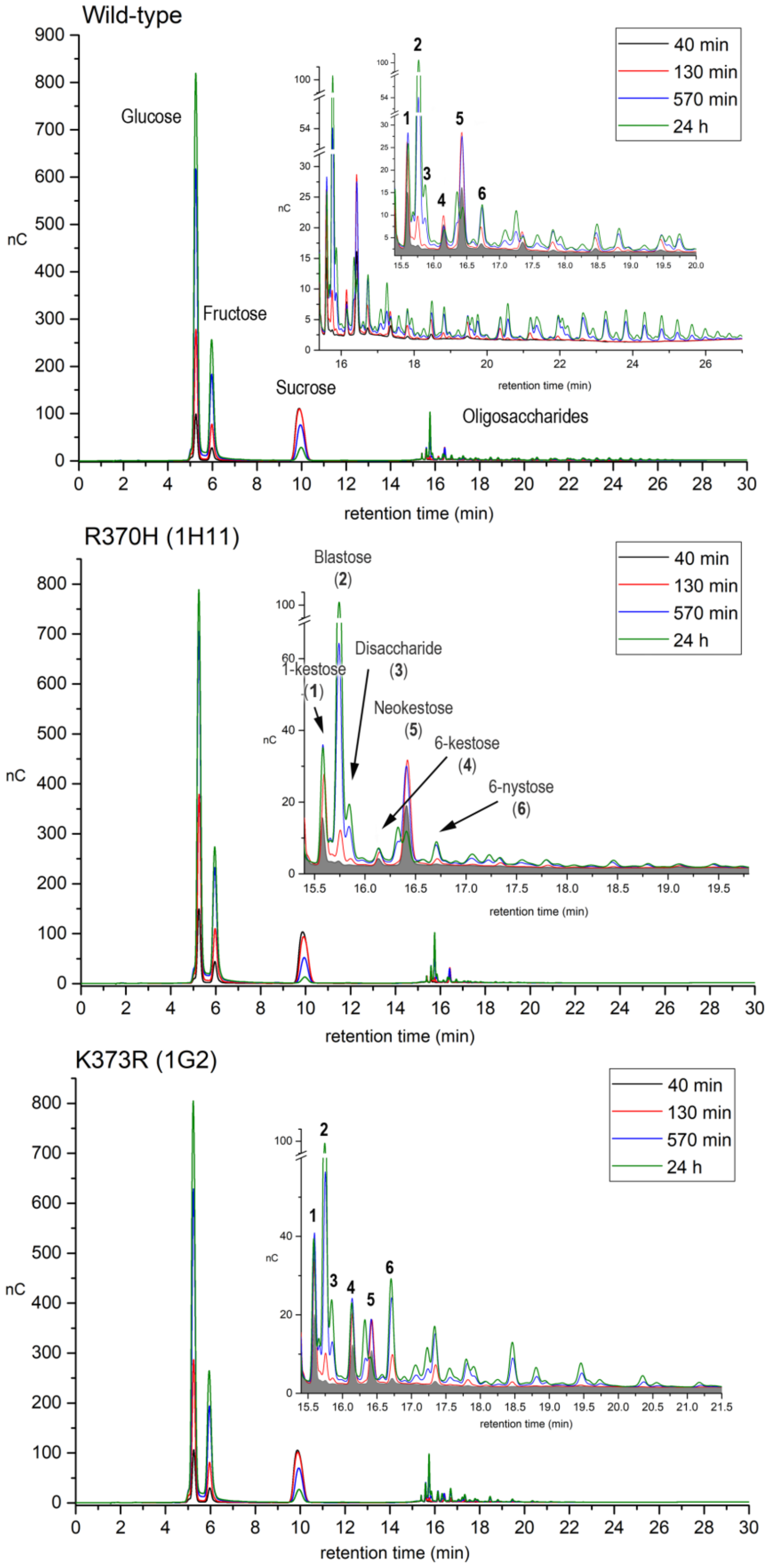

Supplementary Figure S7. Time course HPAEC-PAC chromatograms showing the evolution of products synthesized by the wild-type levansucrase and variants 1H11 and 1G2 over 24 h. Chromatograms corresponding to 40 min reaction samples are shown as a filled area under the curve.

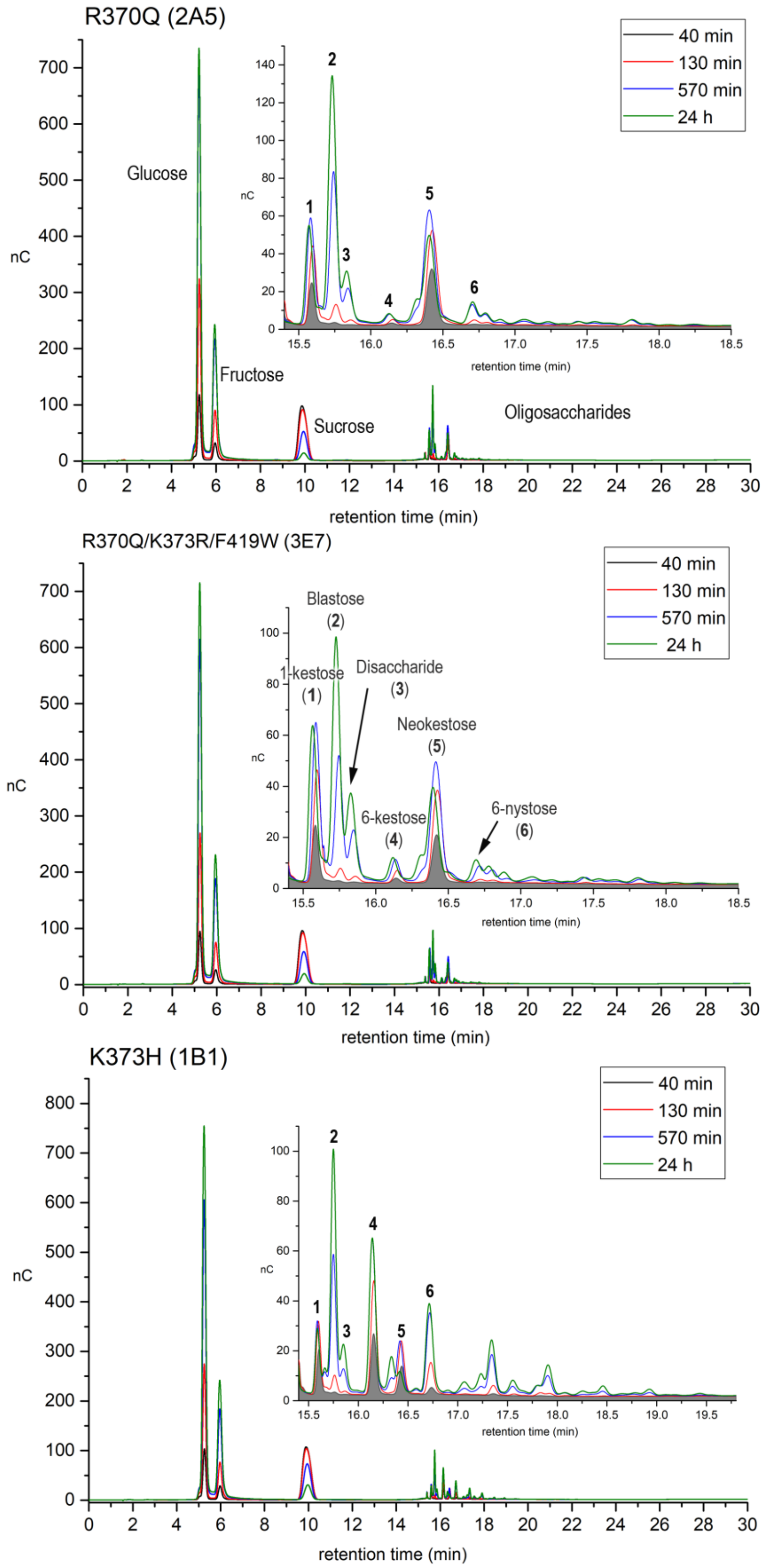

Supplementary Figure S8. Time course HPAEC-PAC chromatograms showing the evolution of products synthesized by variants 2A5, 3E7 and 1B1. Chromatograms corresponding to 40 min reaction samples are shown as a filled area under the curve.

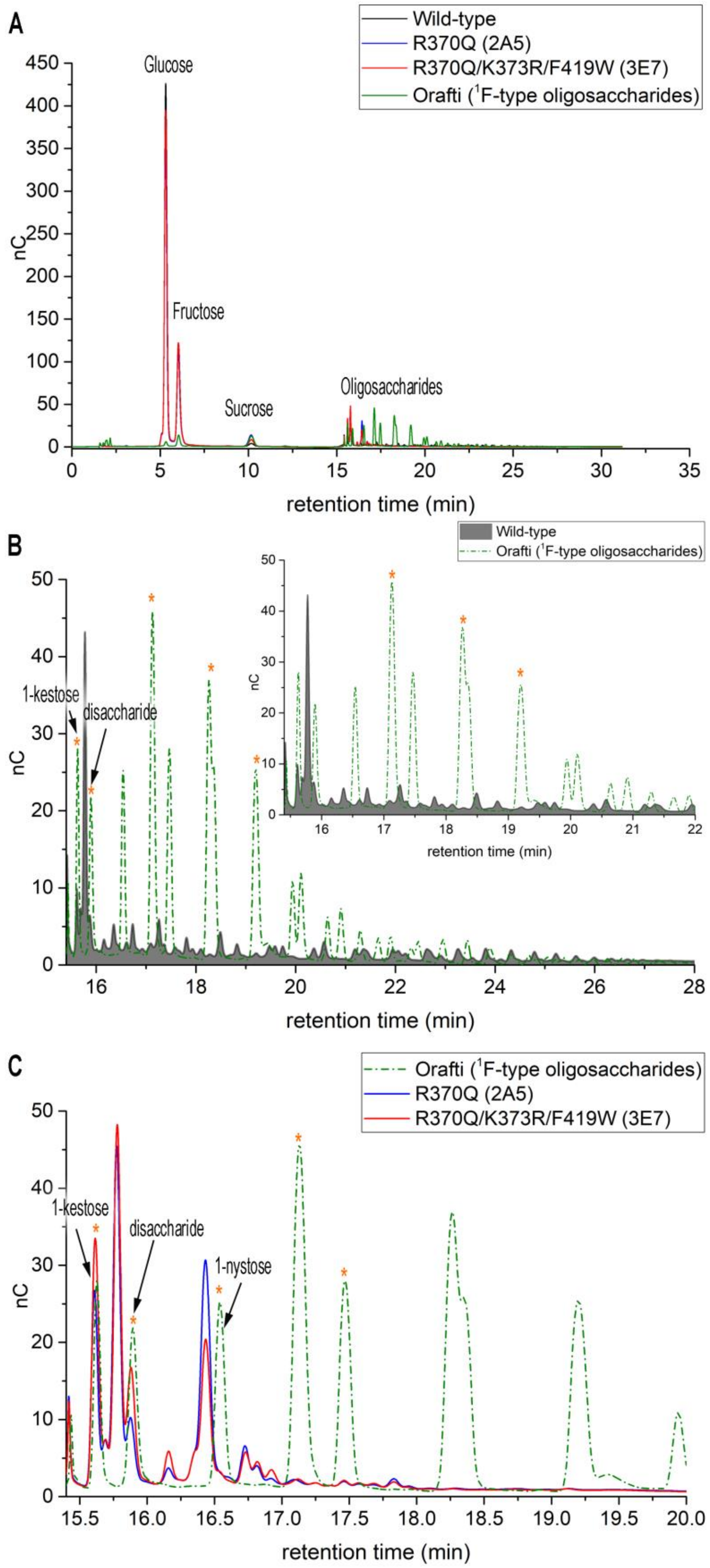

Supplementary Figure S9. Comparison of the HPAEC-PAC product profile of wild-type levansucrase and variants 2A5 and 3E7 with ^{1}F-type oligosaccharides (Orafti®HP). A) Full-length chromatograms. B-C) A closer examination of the oligosaccharide profile of the wild-type levansucrase and variants 2A5 and 3E7. An asterisk indicates the oligosaccharides produced by levansucrases that show the same retention time as ^{1}F-type products. Reactions were performed in Sörensen buffer containing 2 U mL^{-1} of each variant and 0.5 M sucrose. Analyzed samples correspond to reactions stopped after 24 h.

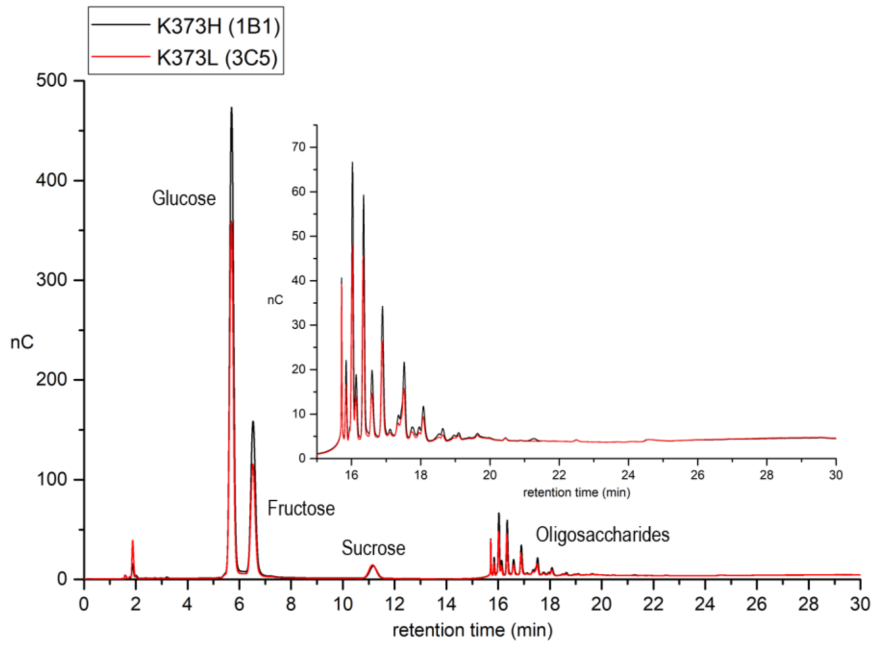

Supplementary Figure S10. Comparison of the HPAEC-PAC product profile of variants K373H and K373L on sucrose.

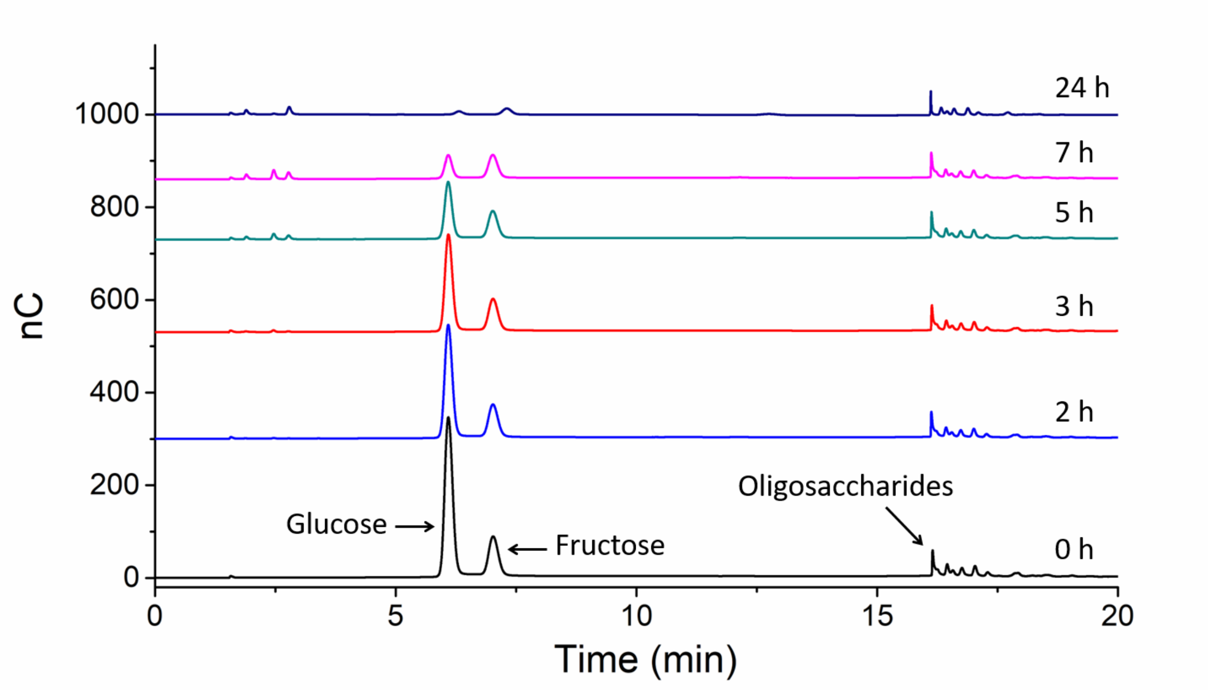

Supplementary Figure S11. HPAEC-PAC analysis of glucose and fructose consumption by Hansenula Polymorpha in a time period of 0 -24 h.

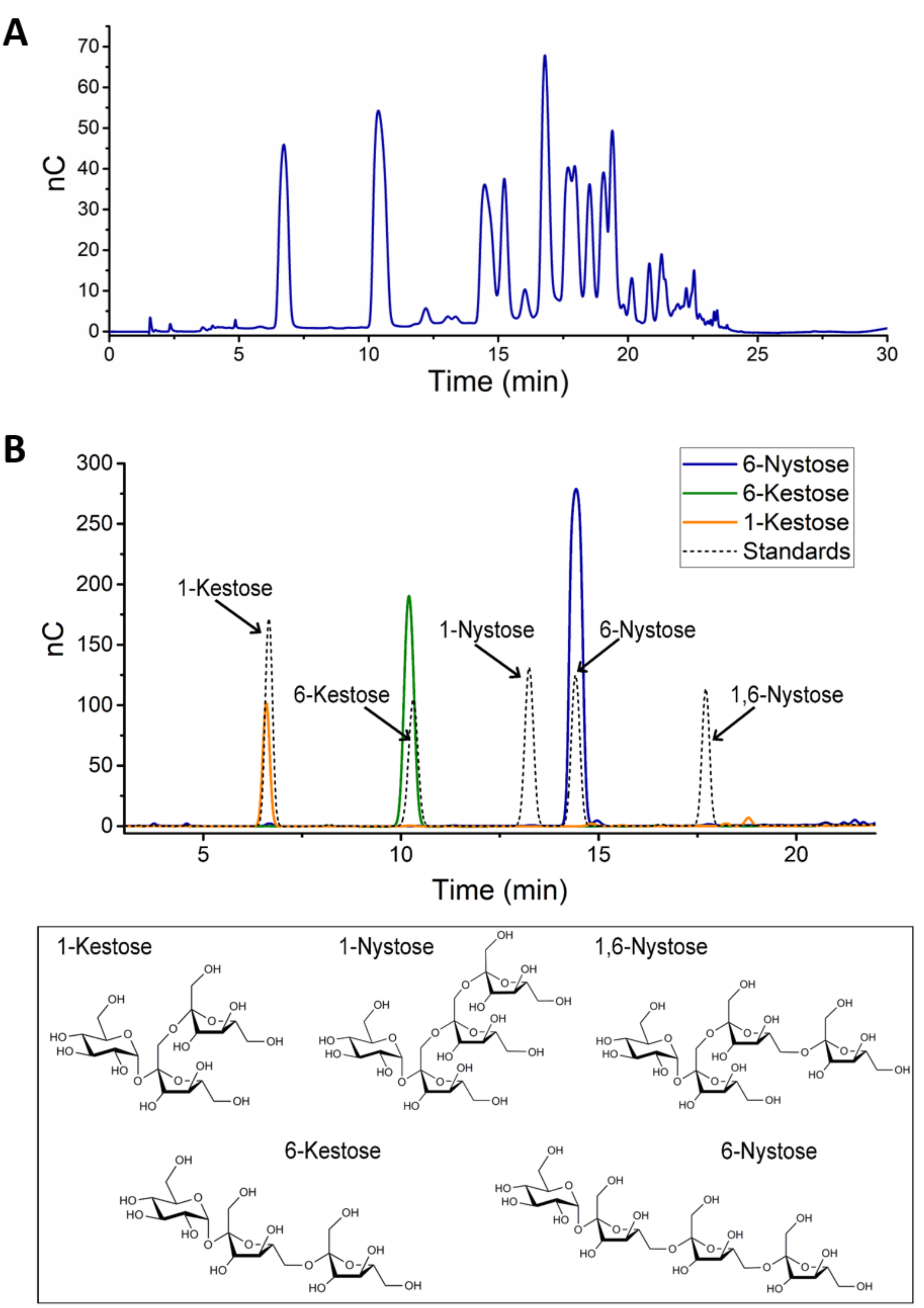

Supplementary Figure S12. Full-length chromatograms of the oligosaccharide analysis shown in the main document as Figure 3. A) One of the fraction of oligosaccharides purified by Bio-Gel ® P2. B) Purified compounds with standards and corresponding chemical structures.

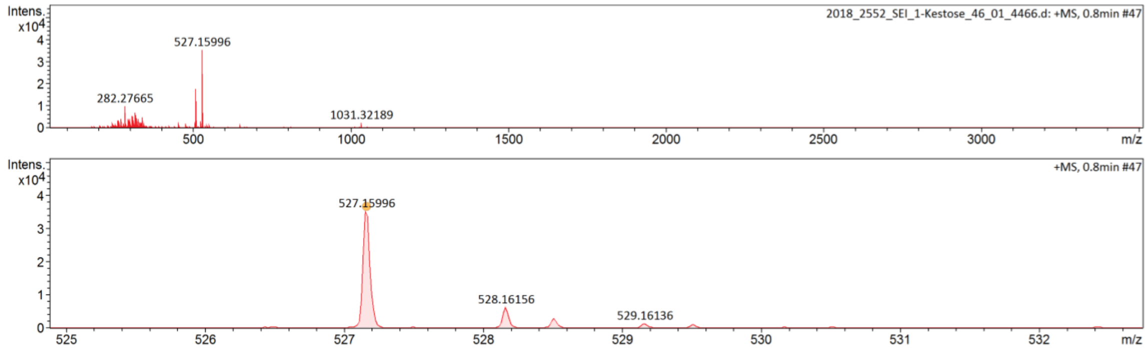

Meas. m/z	#	Ion Formula	m/z	err [ppm]	mSigma	# mSigma	Score	rdb	e¯ Conf	N-Rule
527.15996	1	C18H32NaO16	527.15826	-3.23	16.7	1	100.00	2.5	even	ok

Figure S13: ESI (pos.)-spectrum of 1-kestose

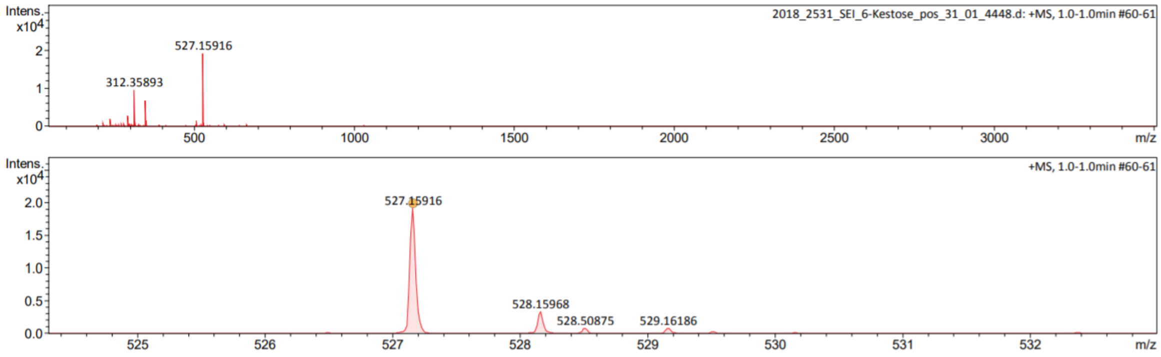

Meas. m/z	#	Ion Formula	m/z	err [ppm]	mSigma	# mSigma	Score	rdb	e⁻ Conf	N-Rule
527.15916	1	C18H32NaO16	527.15826	-1.71	17.7	1	100.00	2.5	even	ok

Figure S14: ESI (pos.)-spectrum of 6-kestose

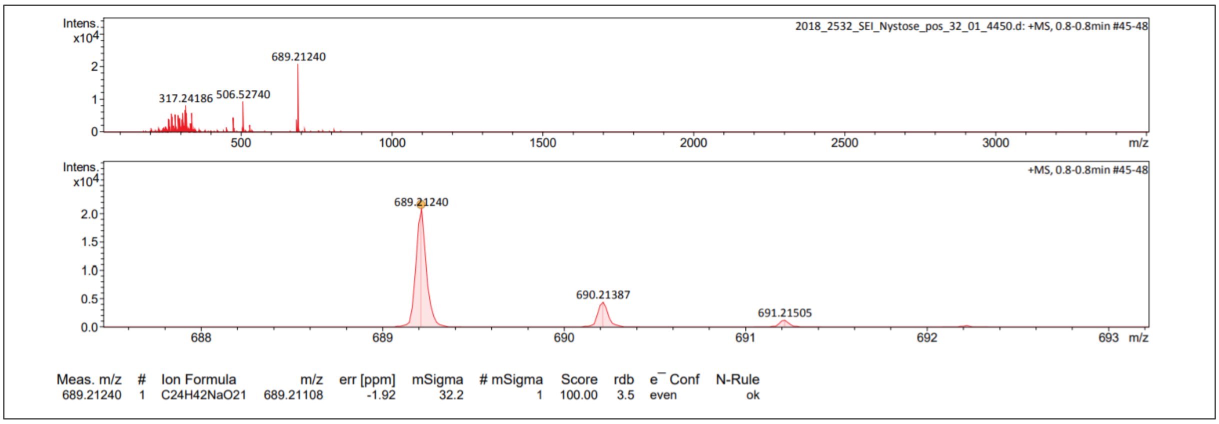

Figure S15: ESI (pos.)-spectrum of 6-Nystose

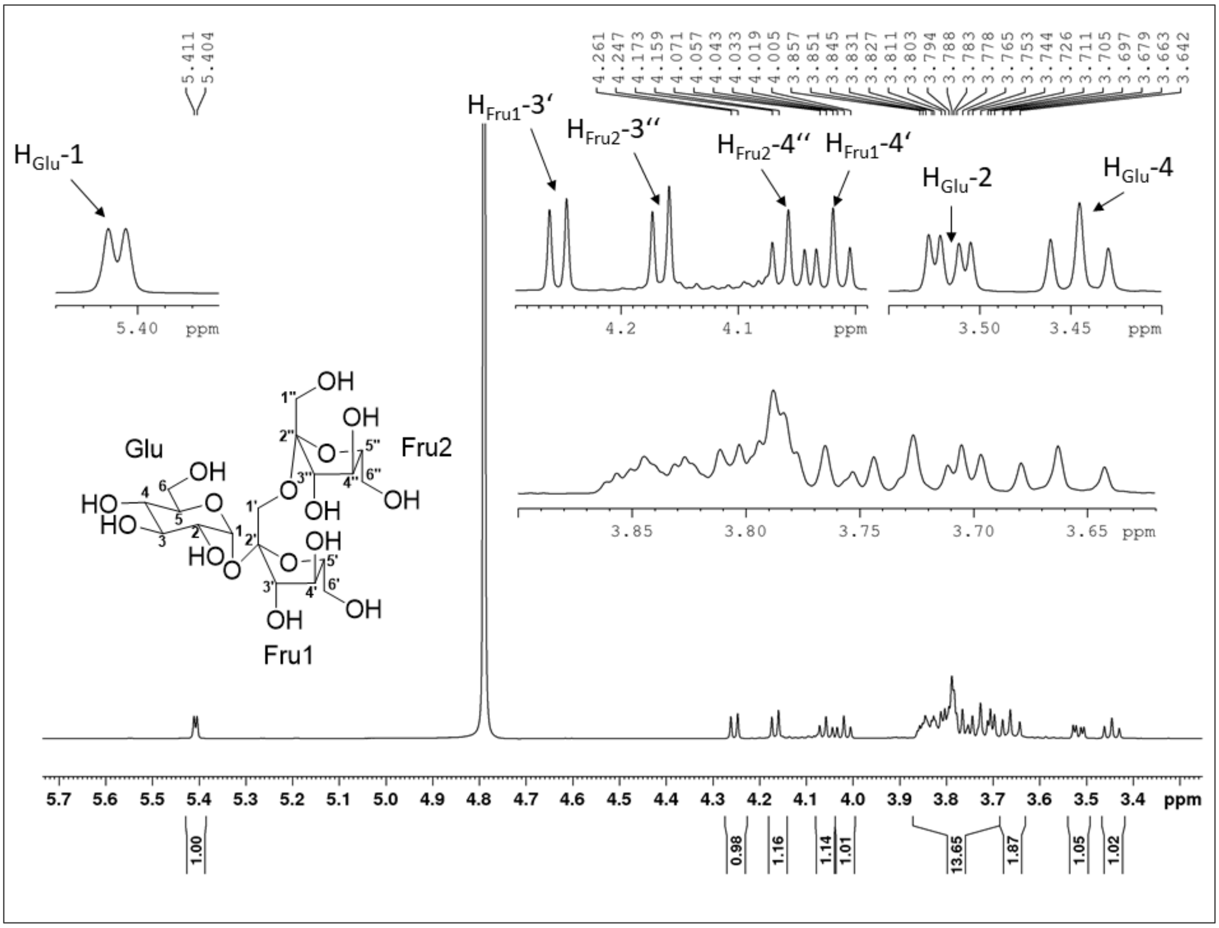

Figure S16: ^{1}H-NMR-spectrum of 1-kestose.

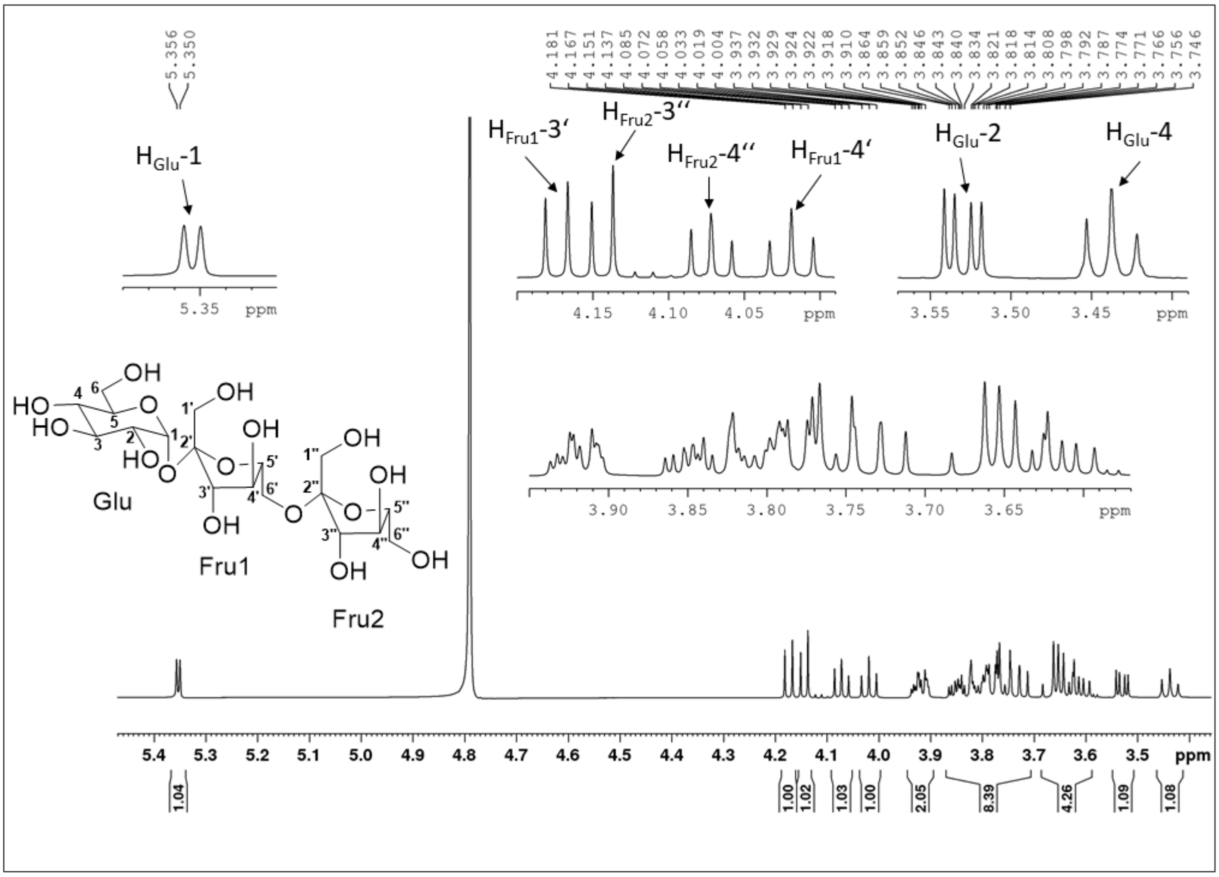

Figure S17: ^{1}H-NMR-spectrum of 6-kestose.

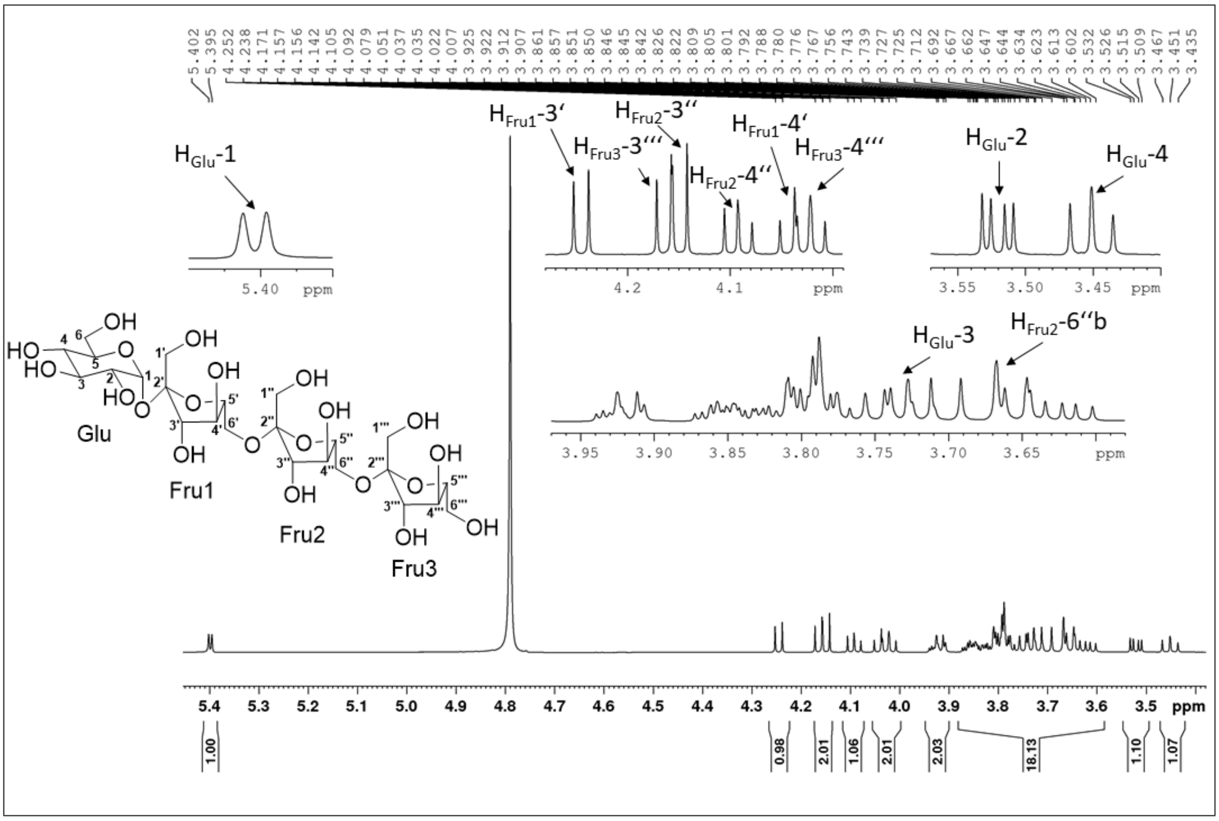

Figure S18: ^{1}H-NMR-spectrum of 6-Nystose.

1 Charoenwongpaiboon, T. *et al.* Modulation of fructooligosaccharide chain length and insight into the product binding motif of *Lactobacillus reuteri* 121 inulosucrase. *Carbohyd Polym* **209**, 111-121, doi:10.1016/j.carbpol.2018.12.078 (2019).

2 Homann, A., Biedendieck, R., Gotze, S., Jahn, D. & Seibel, J. Insights into polymer versus oligosaccharide synthesis: mutagenesis and mechanistic studies of a novel levansucrase from *Bacillus megaterium*. *Biochemical Journal* **407**, 189-198, doi:Doi 10.1042/Bj20070600 (2007).

3 Pijning, T. *et al.* Crystal structure of inulosucrase from *Lactobacillus*: Insights into the substrate specificity and product specificity of GH68 fructansucrases. *J Mol Biol* **412**, 80-93, doi:10.1016/j.jmb.2011.07.031 (2011).

4 Strube, C. P. *et al.* Polysaccharide synthesis of the levansucrase SacB from *Bacillus megaterium* is controlled by distinct surface motifs. *The Journal of Biological Chemistry* **286**, 17593-17600, doi:M110.203166 [pii]

10.1074/jbc.M110.203166 (2011).

5 Pettersen, E. F. *et al.* UCSF chimera - A visualization system for exploratory research and analysis. *J Comput Chem* **25**, 1605-1612, doi:10.1002/jcc.20084 (2004).
